# Supplementary material for: Self-inactivating, all-in-one AAV vectors for precision Cas9 genome editing via homology-directed repair in vivo
Source: Nat Commun. 2021 Nov 1;12:6267. doi: 10.1038/s41467-021-26518-y (PMC8560862; doi:10.1038/s41467-021-26518-y)
Supplement: Supplementary file 1 — Supplementary Information [file 41467_2021_26518_MOESM1_ESM.pdf]

Supplementary Figures

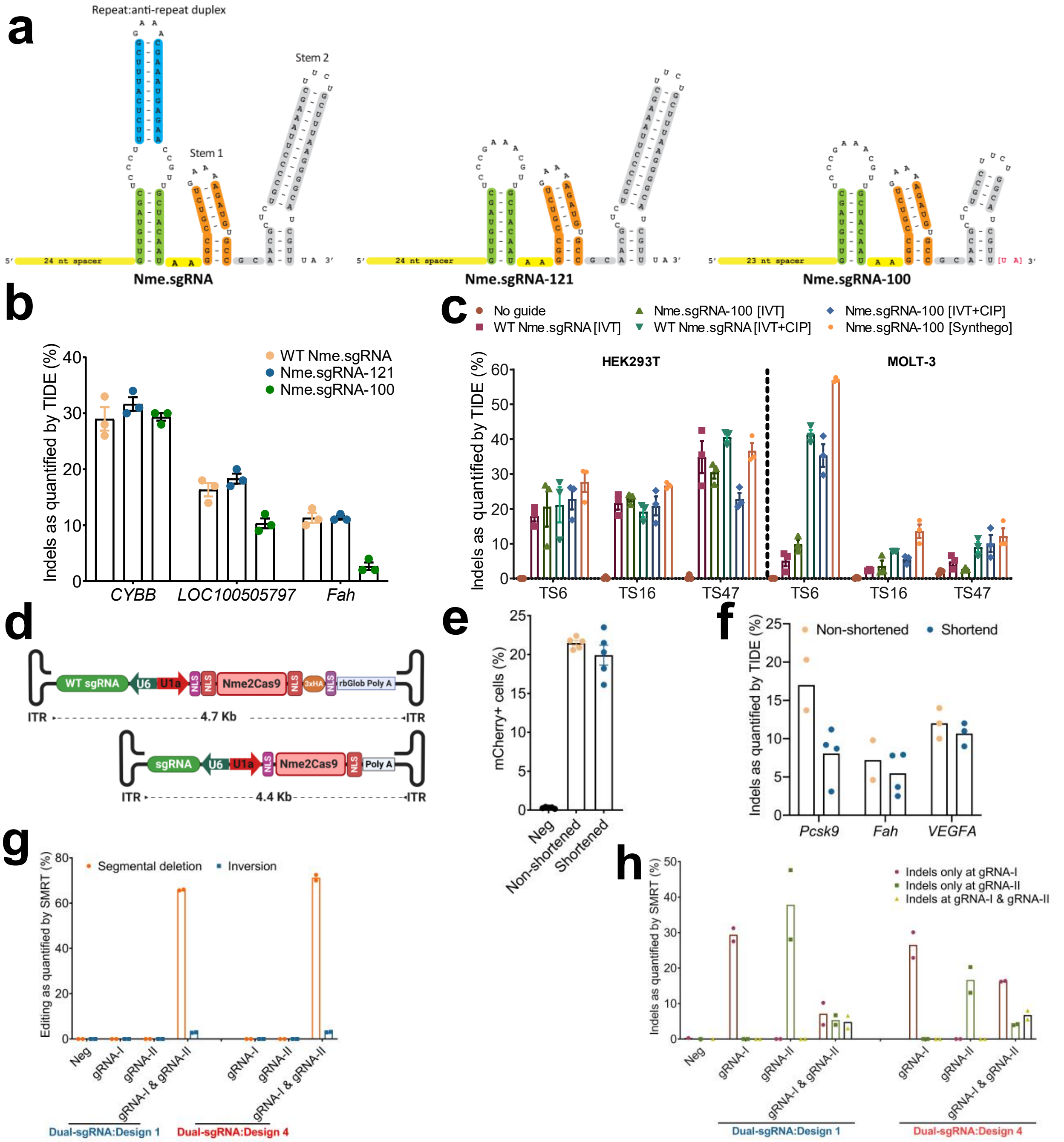

Supplementary Fig. 1 | AAV:Nme2Cas9 vector minimization and dual-sgRNA AAV:Nme2Cas9 plasmids validation. **a**, Illustration of the 145-nt, full-length Nme.sgRNA, the truncated 121-nt Nme.sgRNA-121 and the further truncated 100-nt Nme.sgRNA-100. **b**, Comparison of editing efficiency with WT Nme.sgRNA, Nme.sgRNA-121 and Nme.sgRNA-100 by plasmid transfection at *CYBB*, *LOC100505797* (in HEK293T) and *Fah* (in Neuro2a) genomic loci as estimated by TIDE analysis. (n = 3 biological replicates). **c**, Comparison of RNP editing efficiencies using full-length Nme.sgRNA and Nme.sgRNA-100, produced as T7 RNA polymerase transcripts (*in vitro* transcribed, IVT) with and without treatment with phosphatase (CIP), along with chemically synthesized, commercial Nme.sgRNA-100 guides. Nme2Cas9 RNPs targeting three genomic sites (TS6, TS16 and TS47) were electroporated into HEK293T and T lymphoblast MOLT-3 cells, and editing was assessed by TIDE analysis. (n = 3 biological replicates). **d**, Schematic of the ~4.7 kb (top) and the minimized ~4.4 kb (bottom) AAV vectors expressing Nme2Cas9 and a 121nt sgRNA. ITR, inverted terminal repeats. Nme2Cas9 includes SV40 and nucleoplasmin NLSs on the N and C termini, respectively. **e**, Comparison of editing efficiencies of the non-shortened 4.7 Kb and the shortened 4.4 Kb AAV:Nme2Cas9 constructs by plasmid transfection in TLR-Multi-Cas-Variant 1 (MCV1) lentivector-transduced HEK293T cells, as measured by flow cytometry. (n = 3 biological replicates) **f**, Comparison of editing efficiencies of the ~4.7 Kb and the shortened ~4.4 Kb AAV:Nme2Cas9 plasmids tested by transfection in Neuro2a cells (*Pcsk9* and *Fah*) and HEK293T (*VEGFA*) and estimated by TIDE analysis. (n = 2 non-shortened and n = 4 shortened biological replicates) **g**, Bar graph displaying the percentages of segmental deletion (orange) and inversion (blue) outcomes following gene editing of *Hpd* after plasmid transfection of Dual-sgRNA:Designs 1 and 4 plasmids in Neuro2a cells, as measured by SMRT sequencing analysis. (n = 2 biological replicates) **h**, Bar graph displaying the percentages of indels detected in full-length SMRT sequencing reads after *Hpd* editing by plasmid transfection in Neuro2a cells. The graph indicates genome reads with indels recorded only at gRNA-I (in red), indels only at gRNA-II in (dark green), and indels at both gRNA-I and gRNA-II (yellow) as measured by SMRT sequence analysis (n = 2 biological replicates). Data are presented as mean values ± s.e.m.

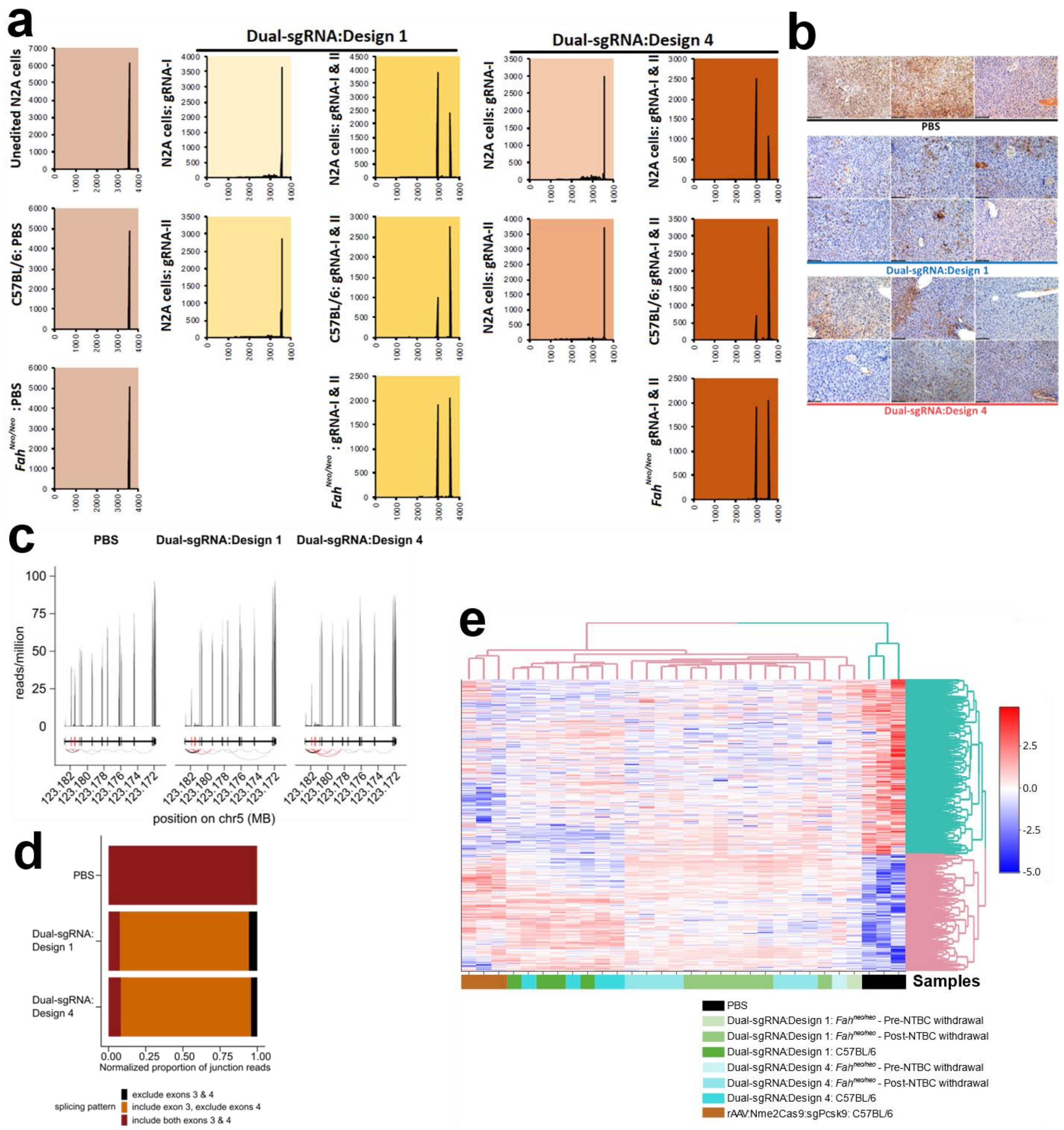

Supplementary Fig. 2 | *In vivo* editing using dual-sgRNA rAAV:Nme2Cas9 vectors. **a**, Representative length distribution plots for mapped SMRT reads indicating the presence of segmental deletions in Neuro2a (N2A) cells after plasmid transfection, or in the livers of C57BL/6 and *Fah*$^{neo/neo}$ mice after AAV8 delivery. **b**, Anti-HPD immunostaining in liver tissues from *Fah*$^{neo/neo}$ mice injected with PBS or with AAV8:Nme2Cas9 Dual-sgRNA:Designs 1 and 4 vectors. Scale bar is 100 µm. **c**, RNA-seq normalized read coverage across *Hpd* exons shows a reduction of exon 4 expression in Dual-sgRNA:Design 1 and Design 4 treated *Fah*$^{neo/neo}$ mice and an increase in exon-exon junction reads skipping exons 3 and/or 4. **d,** Proportions of exon-exon junction reads from RNA-seq data that support the inclusion of both exons 3 and 4 (red), inclusion of only exon 3 (orange), and exclusion of both exons 3 and 4 (black) of *Hpd* in PBS-, Dual-sgRNA:Design 1-, and Dual-sgRNA:Design 4-treated *Fah*$^{neo/neo}$ mice. **e,** Hierarchical cluster analysis of the differentially expressed genes shows the transcriptome differences in the livers of the treated and untreated mice. Mice cohort color coding is indicated in figure legend. Red and blue color of heatmap represent upregulated and downregulated genes, respectively.

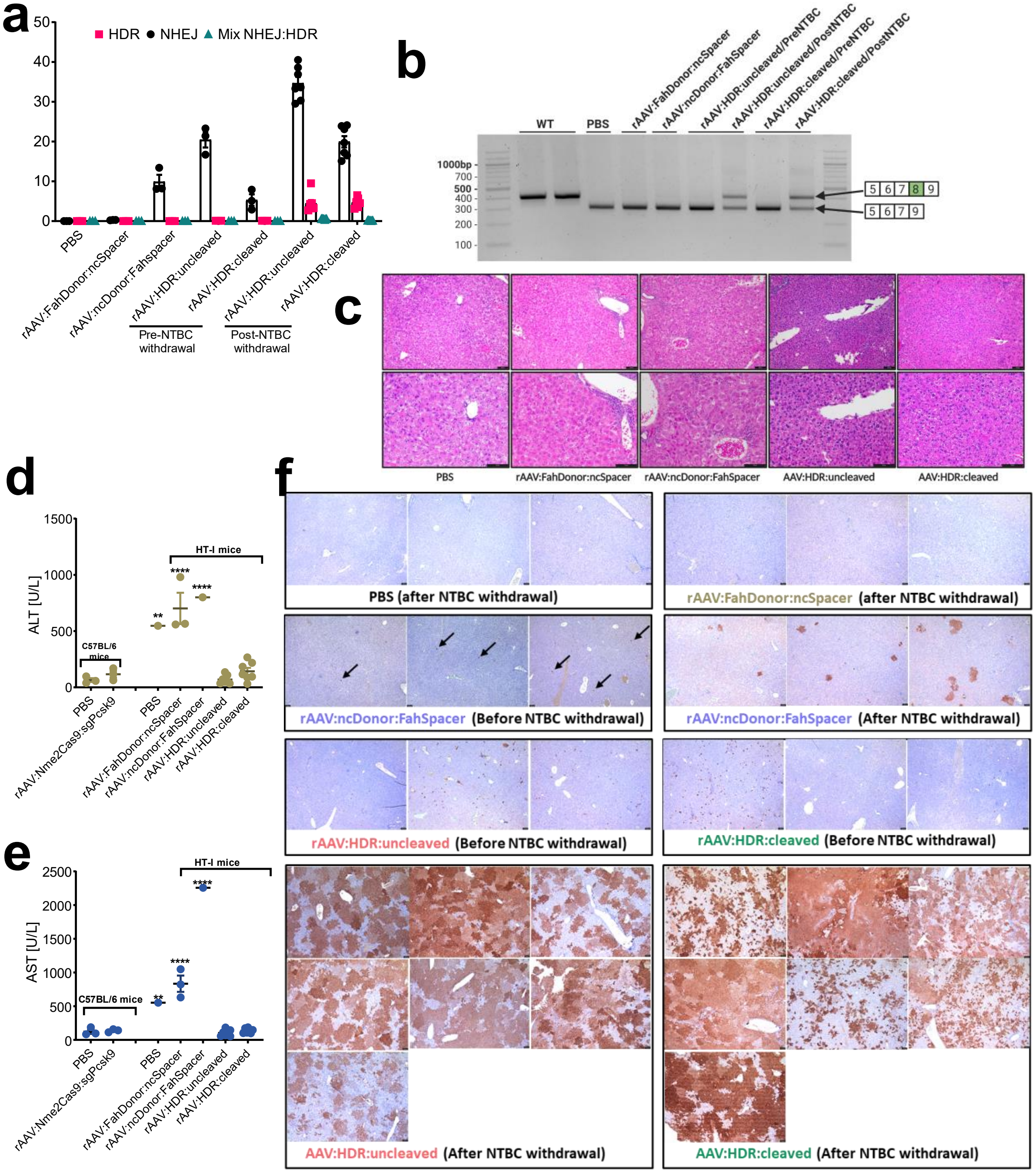

Supplementary Fig. 3 | Improved molecular and pathological phenotypes of HT-I mice after treatment with rAAV:HDR vectors. **a**, Bar graph showing the percentages of NHEJ, HDR and imprecise NHEJ:HDR mix at the *Fah* editing site in livers of HT-I mice pre- and post-NTBC withdrawal, as measured by NGS sequencing of PCR amplicons. **b**, Agarose gel image showing the detection of RT-PCR products of *Fah* mRNA in liver lysate. The lower band (~300 bp) is from products with exon 8 skipped, while the ~400 bp band is from products that include exon 8. **c**, Representative H&E staining of liver from HT-I mice injected with PBS or negative control (rAAV:FahDonor:ncSpacer and rAAV:ncDonor:FahSpacer) vectors, or with rAAV:HDR:cleaved and -uncleaved vectors. Scale bars are 100 μm for the upper panels and 20 mm for the lower panels. **d**, Serum ALT activity in WT C57BL/6 mice injected with PBS and rAAV:Nme2Cas9 vector targeting *Rosa26* from a previous study, in comparison to untreated negative control and treated $Fah^{PM/PM}$ mice. Statistical analysis used one-way ANOVA ($p < 0.0001$) with Dunnett's test. Data are presented as mean values ± s.e.m. **$p = 0.0025$, ****$p < 0.0001$ vs. PBS-injected C57BL/6 group. **e**, Serum AST activity in WT C57BL/6 mice injected with PBS and rAAV:Nme2Cas9 vector targeting *Rosa26* from a previous study, in comparison to untreated negative control and treated $Fah^{PM/PM}$ mice. Statistical analysis used one-way ANOVA ($p<0.0001$) with Dunnett's test. Data are presented as mean values ± s.e.m. **$p = 0.0011$, ****$p < 0.0001$ vs PBS-injected C57BL/6 group. **f**, Anti-FAH immunostaining in liver tissues of all negative-control and treated cohorts. Vectors and drug regimens are indicated for each cohort. Scale bar, 100 μm. Sample size in panels **a**, **d**, and **e**: (n = 3 in PBS, rAAV:FahDonor:ncSpacer, rAAV:ncDonor:FahSpacer and pre-NTBC withdrawal rAAV:HDR:cleaved and -uncleaved cohorts; n = 7 in post-NTBC withdrawal rAAV:HDR:cleaved and -uncleaved cohorts). Panels **b**, **c**, and **f**: The analyses were done once on all mice in each cohort.

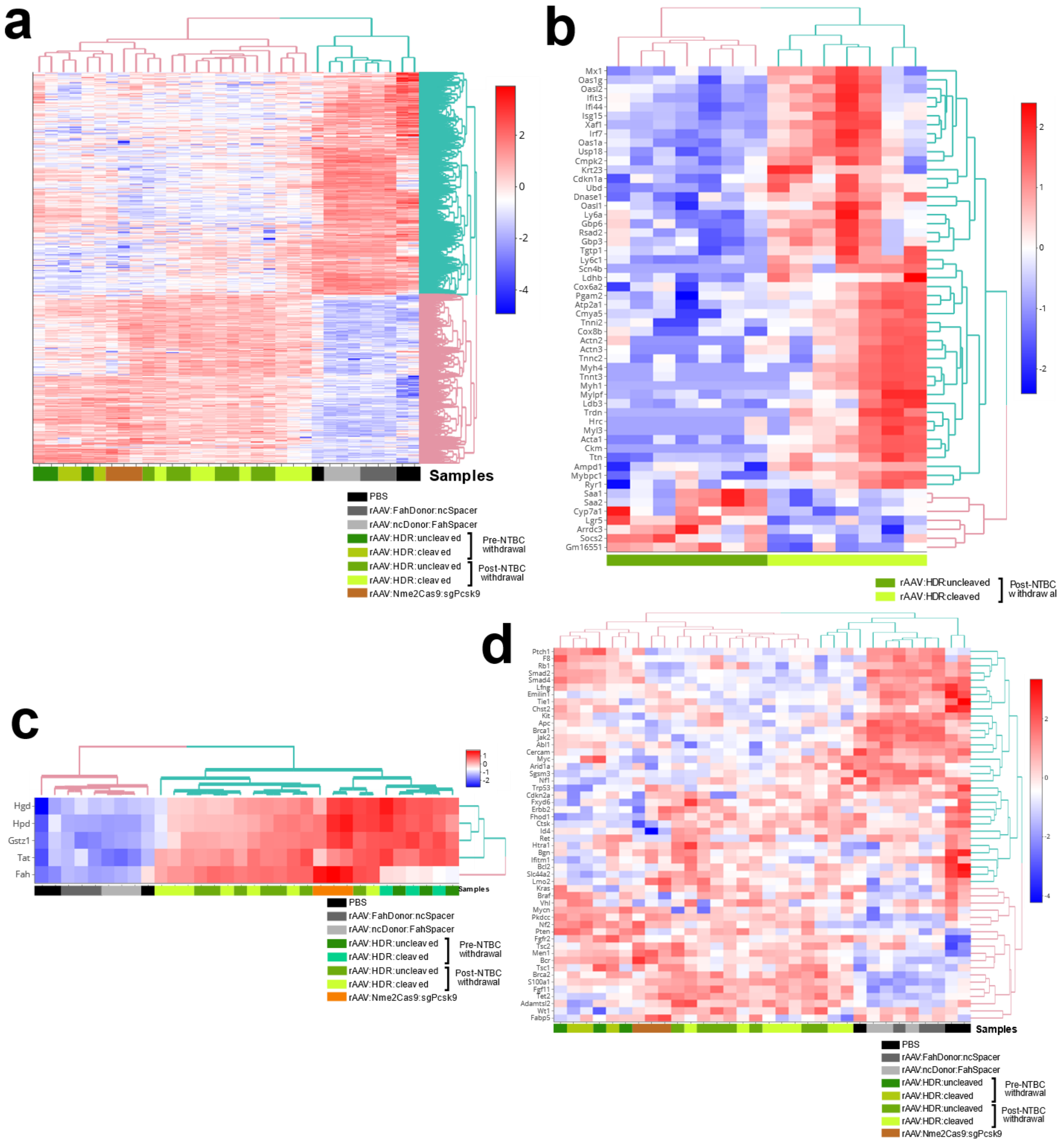

Supplementary Fig. 4 | RNA sequencing analysis showing restoration of healthy transcript expression profiles in *Fah*$^{PM/PM}$ mice after rAAV:HDR:uncleaved and rAAV:HDR:cleaved treatment. **a,** Hierarchical cluster analysis of the differentially expressed genes shows the transcriptome differences in the livers of the treated and untreated mice. **b,** Heatmaps of the global differentially expressed genes between the rAAV:HDR:uncleaved and rAAV:HDR:cleaved cohorts only. **c,** Heatmap of the differentially expressed genes of the tyrosine metabolism pathway. **d,** Heatmap of the differentially expressed known oncogenes and tumor suppressors. Color coding in figure legends indicates treatment regimens. Red and blue color of heatmap represent upregulated and downregulated genes, respectively.

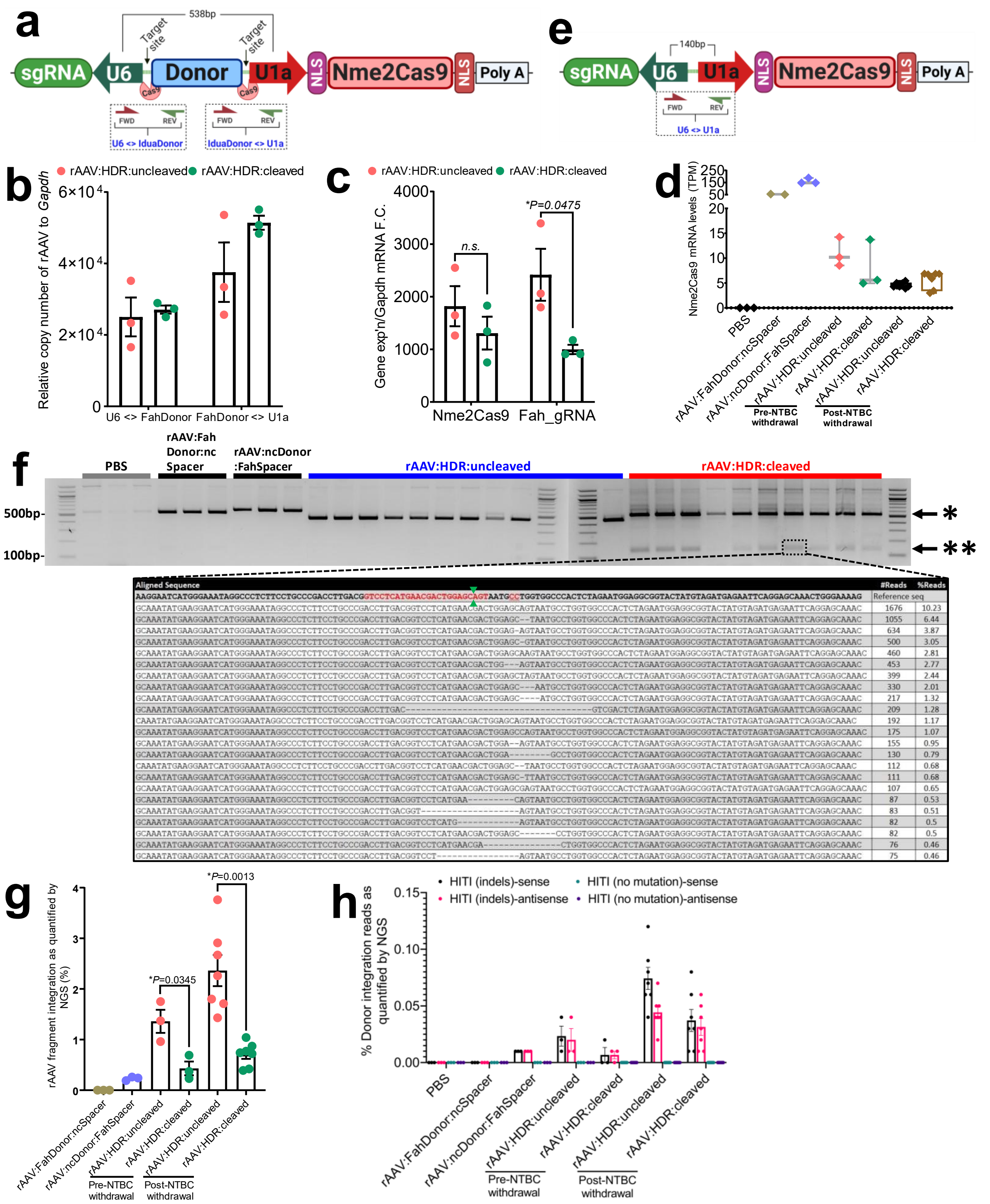

Supplementary Fig. 5 | Reduced rAAV copy number, Nme2Cas9 mRNA expression, and sgRNA expression in HT-I mice treated with rAAV:HDR:cleaved vectors. **a**, Schematic of qPCR assays to quantitate rAAV genome segment copy number in HT-I mouse liver samples. Primer pairs [U6<>FahDonor] and [FahDonor<>U1a] are indicated underneath **b**, rAAV copy numbers in HT-I mouse liver tissues. rAAV copies are unchanged before NTBC withdrawal. **c**, qRT-PCR analyses with total RNA showing reduced sgRNA expression in the rAAV:HDR:cleaved cohort before NTBC withdrawal, while reduced Nme2Cas9 mRNA does not reach statistical significance. **d,** Steady-state mRNA levels from RNA-seq (polyA-enriched, transcripts per million) of Nme2Cas9 mRNA in the liver of *Fah*$^{PM/PM}$ mice. **e**, Schematic of the expected rAAV:HDR:cleaved backbone re-ligated after donor excision. Primer pair [U6<>U1a] is indicated underneath. **f**, Agarose gel indicated the re-ligation of rAAV:HDR:cleaved backbone after donor excision (**) compared to backbone before donor excision (*). NGS data confirms the formation of the ligated junction as indicated in the table. Generated target site is highlighted in red, with N4CC PAM is underlined and Nme2Cas9 cut site is indicated with green arrowheads. This analysis was done once with samples from each mouse in the cohort. **g**, Reduced fragment integration of rAAV in the *Fah* locus in cohorts injected with the rAAV:HDR:cleaved vector, as detected by NGS analysis. **h,** Minimal NHEJ-based integration of the donor is observed at the *Fah* locus in the sense and anti-sense directions, as detected by NGS analysis. Data are presented as mean values ± s.e.m. (n = 3 in PBS, rAAV:FahDonor:ncSpacer, rAAV:ncDonor:FahSpacer and pre-NTBC withdrawal rAAV:HDR:cleaved and -uncleaved cohorts; n = 7 in post-NTBC withdrawal rAAV:HDR:uncleaved and -uncleaved cohorts). *p* values are calculated using Student's t-test (2-sided).

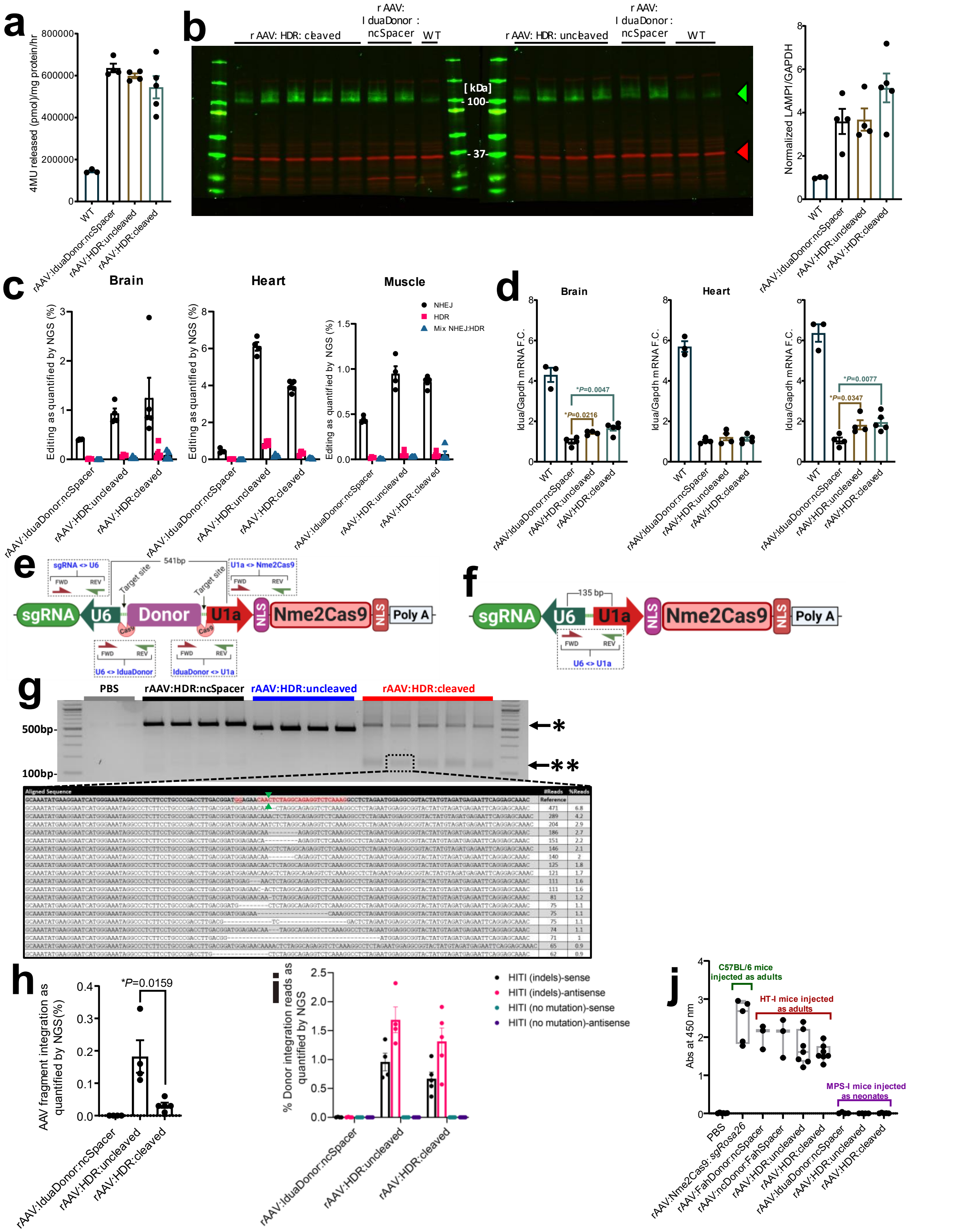

Supplementary Fig. 6 | Reduced rAAV copy number, Nme2Cas9 mRNA expression, and sgRNA expression in MPS-I mice treated with rAAV:HDR:cleaved vectors. a, There is no change in the level of D-hexosaminidase specific activity in liver lysates of treated rAAV:HDR uncleaved/cleaved cohorts compared to rAAV:IduaDonor:ncSpacer mice (negative control). **b,** There is no change in the level of LAMP-1 marker in the MPS-I after treatment as detected by western blot (left). Band intensity was quantified by Image Studio Lite in the bar graph (right). **c**, Bar graph showing the percentage of NHEJ, HDR and imprecise NHEJ:HDR mix at *Idua* in the brain, heart and muscle as measured by NGS sequencing of PCR amplicons from genomic DNA. **d**, qRT-PCR data showing increase in the relative *Idua* mRNA in the brain and muscle tissues as normalized to *Gapdh* mRNA. **e**, Schematic of qPCR assays to quantitate rAAV genome segment copy number in MPS-I mouse liver samples. Primer pairs [sgRNA<>U6], [U6<>IduaDonor], [IduaDonor<>U1a] and [U1a<>Nme2Cas9] are indicated underneath. **f**, Schematic of the expected rAAV:HDR:cleaved backbone re-ligated after donor excision. Primer pair [U6<>U1a] is indicated underneath. **g**, Agarose gel indicated the re-ligation of rAAV:HDR:cleaved backbone after donor excision (**) compared to backbone before donor excision (*). NGS data confirms the formation of the ligated junction as indicated in the table. Generated target site is highlighted in red, with N4CC PAM is underlined and Nme2Cas9 cut site is indicated with green arrowheads. **h**, Reduced fragment integration of rAAV vectors in the *Idua* locus in livers of cohorts injected with the rAAV:HDR:cleaved vector, as detected by NGS analysis. **i,** Level of NHEJ-based integration of the donor observed at the *Idua* locus in the sense and anti-sense directions, as detected by NGS analysis. **j**, Humoral IgG1 immune response to Nme2Cas9 *in vivo* is significantly reduced in rAAV-treated, neonate-injected mice compared to adult-injected cohorts. Data in panels **a-d** and **h-j** are presented as mean values ± s.e.m. (n = 3 mice in WT cohort, n = 4 in rAAV:IduaDonor:ncSpacer and rAAV:HDR:uncleaved cohorts, and n = 5 in rAAV:HDR:uncleaved cohort). *p* values are calculated using Student's t-test (2-sided). Panels **b** and **g**: the analyses were done once with samples from each mouse in the cohort.

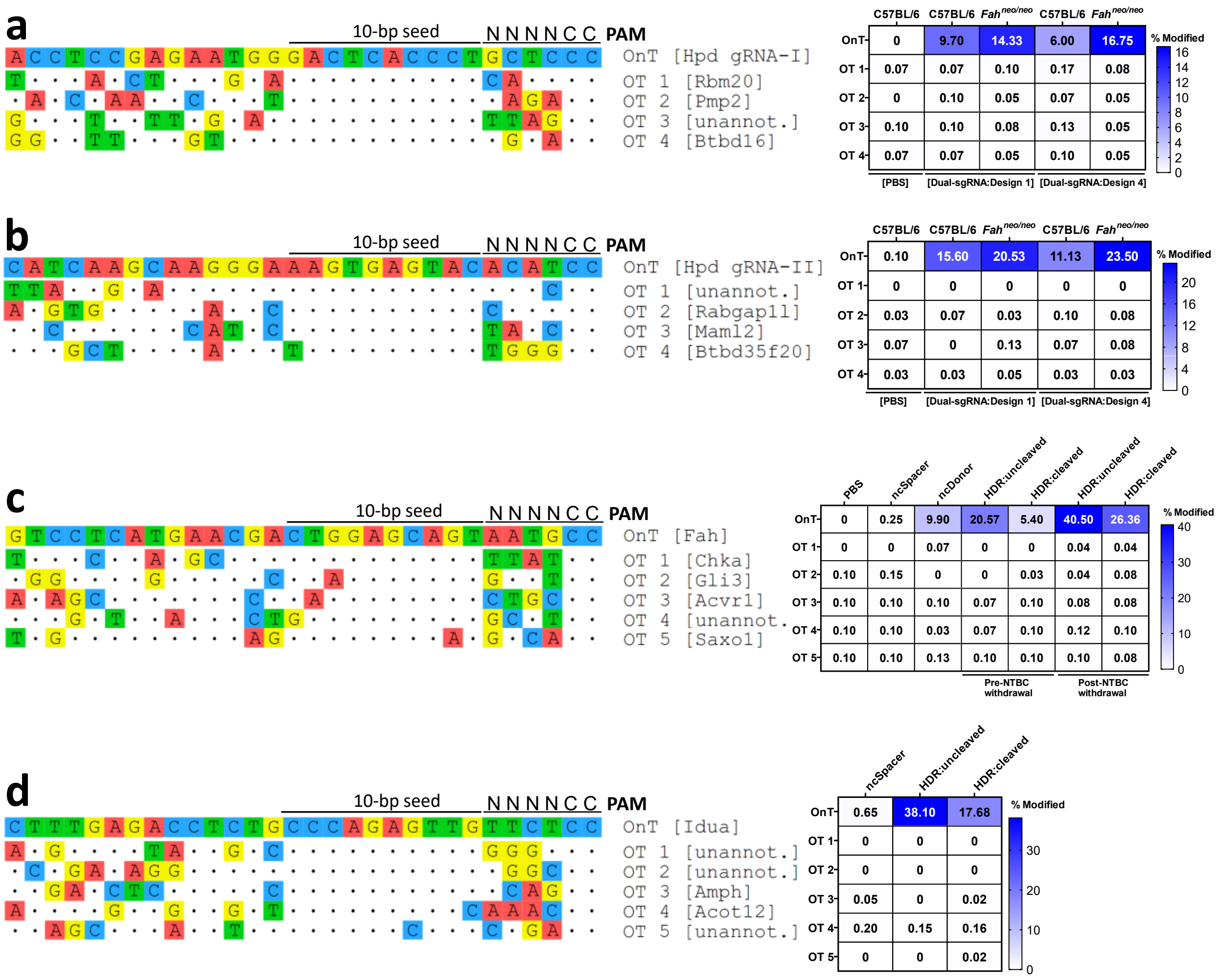

Supplementary Fig. 7 | Nme2Cas9 is a highly specific nuclease with undetectable levels of editing at off-target sites. NGS targeted sequencing for editing at the top potential off-target sites as detected by CRISPRseek analysis for *Hpd* gRNA-I (a) *Hpd* gRNA-II (b) *Fah* (c) and *Idua* (d). The screened sites contained Nme2Cas9 N_4CC PAM and a matching seed sequence to the on-target site. Data are presented as mean values (n = 3-7 biological replicates) using genomic DNA from mice livers according to the treatment indicated on the heatmap.

Supplementary Notes

Nucleotide sequence of all plasmids

pEJS1089 mini-AAV.sgRNA.Nme2Cas9 construct

Legend: ITR **sgRNA scaffold** **GUIDE sequence** *U6 promoter* *U1a promoter* NLS **hNme2Cas9** NLS
short-polyA

cctgcaggcagctgcgcgctcgctcgctcactgaggccgcccgggcaaagcccgggcgtcgggcgacctttggtcgcccggcctcagtgagcgagcgagcgcgcagagagggagtggc
caactccatcactaggggttcctgcggcctctaga**GTTTAAACAAAAAAATAAACGATGCCCCTTAAAGCAGAAGCTTTAAGGGGCAGAGCGTTGCGGC**
ACATCTTTTCAGACGGCCTTATTGTAGCAACGTTTCGGGAGCTACAACNNNNNNNNNNNNNNNNNNNNNNNNC*GGTGTTTCGTCCTTTCCA*
CAAGATATATAAAGCCAAGAAATCGAAATACTTTCAAGTTACGGTAAGCATATGATAGTCCATTTTAAAACATAATTTTAAAACTGCAAACTAC
CCAAGAAATTATTACTTTCTACGTCACGTATTTTGTACTAATATCTTTGTGTTTACAGTCAAATTAATTCTAATTATCTCTCTAACAGCCTTGTATC
*GTATATGCAAATATGAAGGAATCATGGGAAATAGGCCCTC*TTCCTGCCCGACCTTGACGTCGACtctaga*atggaggcggtactatgtagatgagaattc*
aggagcaaactgggaaaagcaactgcttccaaatatttgtgatttttacagtgtagttttggaaaaactcttagcctaccaattcttctaagtgttttaaaatgtgggagccagtac
*acatgaagttatagagtgttttaatgaggcttaaatatttaccgtaactatgaaatgctacgcatatcatgctgttcaggctccgtggccacgcaactcatact*taagcagacagtgg
ttcaaagtttttttcttccattTCAGGTGTCGTGAACACCGCCACCATGGTGCctaagaagaagagaaaggtggaagatATGGCCGCCTTCAAGCCTAACCCaat
caattacatcctgggactggacatcggaatcgcatccgtgggatgggctatggtggagatcgacgaggaggagaatcctatccggctgatcgatctgggcgtgagagtgtttgagaggg
ccgaggtgccaaagaccggcgattctctggctatggcccggagactggcacggagcgtgaggcgcctgacacggagaagggcacacaggctgctgagggcacgccggctgctgaaga
gagagggcgtgctgcaggcagcagacttcgatgagaatggcctgatcaagagcctgccaaacacccccctggcagctgagagcagccgccctggacaggaagctgacaccactggagt
ggtctgccgtgctgctgcacctgatcaagcaccgcggctacctgagccagcggaagaacgagggagagacagcagacaaggagctgggcgccctgctgaagggagtggccaacaatg
cccacgccctgcagaccggcgatttcaggacacctgccgagctggccctgaataagtttgagaaggagtccggccacatcagaaaccagaggggcgactatagccacaccttctcccgc
aaggatctgcaggccgagctgatcctgctgttcgagaagcagaaggagtttggcaatccacacgtgagcggaggcctgaaggagggaatcgagaccctgctgatgacacagaggcctg
ccctgtccggcgacgcagtgcagaagatgctgggacactgcaccttcgagcctgcagagccaaaggccgccaagaacacctacacagccgagcggtttatctggctgacaaagctgaa
caatctgagaatcctggagcagggatccgagaggccactgaccgacacagagagggccaccctgatggatgagccttaccggaagtctaagctgacatatgcccaggccagaaagctg
ctgggcctggaggacaccgccttctttaagggcctgagatacggcaaggataatgccgaggcctccacactgatggagatgaaggcctatcacgccatctctcgcgccctggagaagga
gggcctgaaggacaagaagtccccccctgaacctgagctccgagctgcaggatgagatcggcaccgccttctctctgtttaagaccgacgaggatatcacaggccgcctgaaggacagg
gtgcagcctgagatcctggaggccctgctgaagcacatctctttcgataagtttgtgcagatcagcctgaaggccctgagaaggatcgtgccactgatggagcagggcaagcggtacga
cgaggcctgcgccgagatctacggcgatcactatggcaagaagaacacagaggagaagatctatctgccccctatccctgccgacgagatcagaaatcctgtggtgctgagggccctgt
cccaggcaagaaaagtgatcaacggagtggtgcgccggtacggatctccagcccggatccacatcgagaccgccagagaagtgggcaagagcttcaaggaccggaaggagatcgag
aagagacaggaggagaatcgcaaggatcgggagaaggccgccgccaagtttagggagtacttccctaactttgtgggcgagccaaagtctaaggacatcctgaagctgcgcctgtacg
agcagcagcacggcaagtgtctgtatagcggcaaggagatcaatctggtgcggctgaacgagaagggctatgtggagatcgatcacgccctgcctttctccagaacctgggacgattctt
ttaacaataaggtgctggtgctgggcagcgagaaccagaataagggcaatcagacaccatacgagtatttcaatggcaaggacaactccagggagtggcaggagttcaaggcccgcgt
ggagacctctagatttcccaggagcaagaagcagcggatcctgctgcagaagttcgacgaggatggctttaaggagtgcaacctgaatgacaccagatacgtgaaccggttcctgtgcc
agtttgtggccgatcacatcctgctgaccggcaagggcaagagaagggtgttcgcctctaatggccagatcacaaacctgctgaggggattttggggactgaggaaggtgcgggcagag
aatgacagacaccacgcactggatgcagtggtggtggcatgcagcaccgtggcaatgcagcagaagatcacaagattcgtgaggtataaggagatgaacgcctttgacggcaagacc
atcgataaggagacaggcaaggtgctgcaccagaagacccacttcccccagccttgggagttctttgcccaggaagtgatgatccgggtgttcggcaagccagacggcaagcctgagtt
tgaggaggccgataccccagagaagctgaggacactgctggcagagaagctgtctagcaggccagaggcagtgcacgagtacgtgaccccactgttcgtgtccagggcacccaatcgg
aagatgtctggcgcccacaaggacacactgagaagcgccaagaggtttgtgaagcacaacgagaagatctccgtgaagagagtgtggctgaccgagatcaagctggccgatctggag
aacatggtgaattacaagaacggcagggagatcgagctgtatgaggccctgaaggcaaggctggaggcctacggaggaaatgccaagcaggccttcgacccaaaggataaccccttttt
ataagaagggaggacagctggtgaaggccgtgcgggtggagaagacccaggagagcggcgtgctgctgaataagaagaacgcctacacaatcgccgacaatggcgatatggtgaga
gtggacgtgttctgtaaggtggataagaagggcaagaatcagtactttatcgtgcctatctatgcctggcaggtggccgagaacatcctgccagacatcgattgcaagggctacagaatt
gacgatagctatacattctgtttttccctgcacaagtatgacctgatcgccttccagaaggatgagaagtccaaggtggagtttgcctactatatcaattgcgactcctctaacggcaggttc
tacctggcctggcacgataagggcagcaaggagcagcagtttcgcatctccacccagaatctggtgctgatccagaagtatcaggtgaacgagctgggcaaggagatcaggccatgtcg
gctgaagAAGCGCCCACCCGTGCGGGAGGATAAGCGGCCCGCAGCAACCAAGAAGGCAGGACAGGCCAAGAAGAAGAAGTAAAATAAAAGA
TCTTTATTTTCATTAGATCTGTGTGTTGGTTTTTTGTGTAAGCgcggccgcaggaacccctagtgatggagttggccactccctctctgcgcgctcgctcgctcactgag
gccgggcgaccaaaggtcgcccgacgcccgggctttgcccgggcggcctcagtgagcgagcgagcgcgcagctgcctgcagg

pEJS1096: Dual-sgRNA.Design 1 construct

Legend: ITR (first: *U6 promoter* **GUIDE sequence** **sgRNA scaffold**) (second: ***U6 promoter*** **GUIDE sequence** **sgRNA scaffold**) *U1a promoter* NLS **hNme2Cas9** NLS short-polyA

cctgcaggcagctgcgcgctcgctcgctcactgaggccgcccgggcaaagcccgggcgtcgggcgacctttggtcgcccggcctcagtgagcgagcgagcgcgcagagagggagtggc
caactccatcactaggggttcctgcggcctctaga*GTTTAAACAAAAAAATAAACGATGCCCCTTAAAGCAGAAGCTTTAAGGGGCAGAGCGTTGCGGCA*
*CATCTTTTCAGACGGCCTTATTGTAGCAACGTTTCGGGAGCTACAAC*TGAAGAGCTGGAATgctcttcCGGTGTTTCGTCCTTTCCACAAGATATA
TAAAGCCAAGAAATCGAAATACTTTCAAGTTACGGTAAGCATATGATAGTCCATTTTAAAACATAATTTTAAAACTGCAAACTACCCAAGAAAT
TATTACTTTCTACGTCACGTATTTTGTACTAATATCTTTGTGTTTACAGTCAAATTAATTCTAATTATCTCTCTAACAGCCTTGTATCGTATATGCA
AATATGAAGGAATCATGGGAAATAGGCCCTCTTCCTGCCCGACCTTGACGGGTAGT***GTTTAAACAAAAAAATAAACGATGCCCCTTAAAGCA***
GAAGCTTTAAGGGGCAGAGCGTTGCGGCACATCTTTTCAGACGGCCTTATTGTAGCAACGTTTCGGGAGCTACAACTgagacgTGGAATcgtct
cCGGTGTTTCGTCCTTTCCACAAGATATATAAAGCCAAGAAATCGAAATACTTTCAAGTTACGGTAAGCATATGATAGTCCATTTTAAAACATA
ATTTTAAAACTGCAAACTACCCAAGAAATTATTACTTTCTACGTCACGTATTTTGTACTAATATCTTTGTGTTTACAGTCAAATTAATTCTAATTA
TCTCTCTAACAGCCTTGTATCGTATATGCAAATATGAAGGAATCATGGGAAATAGGCCCTCATTATCCTCTAGA*ATGGAGGCGGTACtatgtaga*
tgagaattcaggagcaaactgggaaaagcaactgcttccaaatatttgtgatttttacagtgtagttttggaaaaactcttagcctaccaattcttctaagtgttttaaaatgtggg
*agccagtacacatgaagttatagagtgttttaatgaggcttaaatatttaccgtaactatgaaatgctacgcatatcatgctgttcaggctccgtggccacgcaactcatact*taag
cagacagtggttcaaagtttttttcttccattTCAGGTGTCGTGAACACCGCCACCATGGTGCctaagaagaagagaaaggtggaagat**ATGGCCGCCTTCAAGCC**
TAACCCaatcaattacatcctgggactggacatcggaatcgcatccgtgggatgggctatggtggagatcgacgaggaggagaatcctatccggctgatcgatctgggcgtgagag
tgtttgagagggccgaggtgccaaagaccggcgattctctggctatggcccggagactggcacggagcgtgaggcgcctgacacggagaagggcacacaggctgctgagggcacgc
cggctgctgaagagagagggcgtgctgcaggcagcagacttcgatgagaatggcctgatcaagagcctgccaaacacccctggcagctgagagcagccgccctggacaggaagct
gacaccactggagtggtctgccgtgctgctgcacctgatcaagcaccgcggctacctgagccagcggaagaacgagggagagacagcagacaaggagctgggcgccctgctgaagg
gagtggccaacaatgcccacgccctgcagaccggcgatttcaggacacctgccgagctggccctgaataagtttgagaaggagtccggccacatcagaaaccagaggggcgactat
agccacaccttctcccgcaaggatctgcaggccgagctgatcctgctgttcgagaagcagaaggagtttggcaatccacacgtgagcggaggcctgaaggagggaatcgagaccctg
ctgatgacacagaggcctgccctgtccggcgacgcagtgcagaagatgctgggacactgcaccttcgagcctgcagagccaaaggccgccaagaacacctacacagccgagcggttt
atctggctgacaaagctgaacaatctgagaatcctggagcagggatccgagaggccactgaccgacacagagagggccaccctgatggatgagccttaccggaagtctaagctgac
atatgcccaggccagaaagctgctgggcctggaggacaccgccttctttaagggcctgagatacggcaaggataatgccgaggcctccacactgatggagatgaaggcctatcacgc
catctctcgcgccctggagaaggagggcctgaaggacaagaagtccccctgaacctgagctccgagctgcaggatgagatcggcaccgccttctctctgtttaagaccgacgaggat
atcacaggccgcctgaaggacagggtgcagcctgagatcctggaggccctgctgaagcacatctctttcgataagtttgtgcagatcagcctgaaggccctgagaaggatcgtgccac
tgatggagcagggcaagcggtacgacgaggcctgcgccgagatctacggcgatcactatggcaagaagaacacagaggagaagatctatctgccccctatccctgccgacgagatc
agaaatcctgtggtgctgagggccctgtcccaggcaagaaaagtgatcaacggagtggtgcgccggtacggatctccagcccggatccacatcgagaccgccagagaagtgggcaa
gagcttcaaggaccggaaggagatcgagaagagacaggaggagaatcgcaaggatcgggagaaggccgccgccaagtttagggagtacttccctaactttgtgggcgagccaaag
tctaaggacatcctgaagctgcgcctgtacgagcagcagcacggcaagtgtctgtatagcggcaaggagatcaatctggtgcggctgaacgagaagggctatgtggagatcgatcac
gccctgcctttctccagaacctgggacgattcttttaacaataaggtgctggtgctgggcagcgagaaccagaataagggcaatcagacaccatacgagtatttcaatggcaaggaca
actccagggagtggcaggagttcaaggcccgcgtggagacctctagatttcccaggagcaagaagcagcggatcctgctgcagaagttcgacgaggatggctttaaggagtgcaacc
tgaatgacaccagatacgtgaaccggttcctgtgccagtttgtggccgatcacatcctgctgaccggcaagggcaagagaagggtgttcgcctctaatggccagatcacaaacctgct
gaggggattttggggactgaggaaggtgcgggcagagaatgacagacaccacgcactggatgcagtggtggtggcatgcagcaccgtggcaatgcagcagaagatcacaagattc
gtgaggtataaggagatgaacgcctttgacggcaagaccatcgataaggagacaggcaaggtgctgcaccagaagacccacttcccccagccttgggagttctttgcccaggaagtg
atgatccgggtgttcggcaagccagacggcaagcctgagtttgaggaggccgataccccagagaagctgaggacactgctggcagagaagctgtctagcaggccagaggcagtgca
cgagtacgtgaccccactgttcgtgtccagggcacccaatcggaagatgtctggcgcccacaaggacacactgagaagcgccaagaggtttgtgaagcacaacgagaagatctccgt
gaagagagtgtggctgaccgagatcaagctggccgatctggagaacatggtgaattacaagaacggcagggagatcgagctgtatgaggccctgaaggcaaggctggaggcctac
ggaggaaatgccaagcaggccttcgacccaaaggataaccccttttataagaagggaggacagctggtgaaggccgtgcgggtggagaagacccaggagagcggcgtgctgctga
ataagaagaacgcctacacaatcgccgacaatggcgatatggtgagagtggacgtgttctgtaaggtggataagaagggcaagaatcagtactttatcgtgcctatctatgcctggca
ggtggccgagaacatcctgccagacatcgattgcaagggctacagaattgacgatagctatacattctgtttttccctgcacaagtatgacctgatcgccttccagaaggatgagaagt
ccaaggtggagtttgcctactatatcaattgcgactcctctaacggcaggttctacctggcctggcacgataagggcagcaaggagcagcagtttcgcatctccacccagaatctggtg
ctgatccagaagtatcaggtgaacgagctgggcaaggagatcaggccatgtcggctgaagAAGCGCCCACCCGTGCGGGAGGATAAGCGGCCCGCAGCAACCA
AGAAGGCAGGACAGGCCAAGAAGAAGAAGTAAAATAAAAGATCTTTATTTTCATTAGATCTGTGTGTTGGTTTTTTGTGTAAGCgcggccgcagg
aacccctagtgatggagttggccactccctctctgcgcgctcgctcgctcactgaggccgggcgaccaaaggtcgcccgacgcccgggctttgcccgggcggcctcagtgagcgagcgag
cgcgcagctgcctgcagg

pEJS1099: Dual-sgRNA.Design 4 construct

Legend: ITR (first: **sgRNA scaffold** **GUIDE sequence** *U6 promoter*) *U1a promoter* NLS **hNme2Cas9** NLS short-polyA (second: **sgRNA scaffold** **GUIDE sequence** ***U6 promoter***)

cctgcaggcagctgcgcgctcgctcgctcactgaggccgcccgggcaaagcccgggcgtcgggcgacctttggtcgcccggcctcagtgagcgagcgagcgcgcagagagggagtggc
caactccatcactaggggttcctgcggcctctaga**GTTTAAACAAAAAAATAAACGATGCCCCTTAAAGCAGAAGCTTTAAGGGGCAGAGCGTTGCGGC**
ACATCTTTTCAGACGGCCTTATTGTAGCAACGTTTCGGGAGCTACAACNNNNNNNNNNNNNNNNNNNNNNNN*GGTGTTTCGTCCTTTCC*
ACAAGATATATAAAGCCAAGAAATCGAAATACTTTCAAGTTACGGTAAGCATATGATAGTCCATTTTAAAACATAATTTTAAAACTGCAAACTA
CCCAAGAAATTATTACTTTCTACGTCACGTATTTTGTACTAATATCTTTGTGTTTACAGTCAAATTAATTCTAATTATCTCTCTAACAGCCTTGTAT
*CGTATATGCAAATATGAAGGAATCATGGGAAATAGGCCCTC*TTCCTGCCCGACCTTGACGTCGACtctaga*atggaggcggtactatgtagatgagaatt*
caggagcaaactgggaaaagcaactgcttccaaatatttgtgatttttacagtgtagttttggaaaaactcttagcctaccaattcttctaagtgttttaaaatgtgggagccagta
*cacatgaagttatagagtgttttaatgaggcttaaatatttaccgtaactatgaaatgctacgcatatcatgctgttcaggctccgtggccacgcaactcatact*taagcagacagtg
gttcaaagtttttttcttccattTCAGGTGTCGTGAACACCGCCACCATGGTGCctaagaagaagagaaaggtggaagat**ATGGCCGCCTTCAAGCCTAACCCa**
atcaattacatcctgggactggacatcggaatcgcatccgtgggatgggctatggtggagatcgacgaggaggagaatcctatccggctgatcgatctgggcgtgagagtgtttgaga
gggccgaggtgccaaagaccggcgattctctggctatggcccggagactggcacggagcgtgaggcgcctgacacggagaagggcacacaggctgctgagggcacgccggctgctg
aagagagagggcgtgctgcaggcagcagacttcgatgagaatggcctgatcaagagcctgccaaacacccccctggcagctgagagcagccgccctggacaggaagctgacaccact
ggagtggtctgccgtgctgctgcacctgatcaagcaccgcggctacctgagccagcggaagaacgagggagagacagcagacaaggagctgggcgccctgctgaagggagtggcc
aacaatgcccacgccctgcagaccggcgatttcaggacacctgccgagctggccctgaataagtttgagaaggagtccggccacatcagaaaccagaggggcgactatagccacac
cttctcccgcaaggatctgcaggccgagctgatcctgctgttcgagaagcagaaggagtttggcaatccacacgtgagcggaggcctgaaggagggaatcgagaccctgctgatgac
acagaggcctgccctgtccggcgacgcagtgcagaagatgctgggacactgcaccttcgagcctgcagagccaaaggccgccaagaacacctacacagccgagcggtttatctggct
gacaaagctgaacaatctgagaatcctggagcagggatccgagaggccactgaccgacacagagagggccaccctgatggatgagccttaccggaagtctaagctgacatatgccc
aggccagaaagctgctgggcctggaggacaccgccttctttaagggcctgagatacggcaaggataatgccgaggcctccacactgatggagatgaaggcctatcacgccatctctcg
cgccctggagaaggagggcctgaaggacaagaagtccccctgaacctgagctccgagctgcaggatgagatcggcaccgccttctctctgtttaagaccgacgaggatatcacagg
ccgcctgaaggacagggtgcagcctgagatcctggaggccctgctgaagcacatctctttcgataagtttgtgcagatcagcctgaaggccctgagaaggatcgtgccactgatggag
cagggcaagcggtacgacgaggcctgcgccgagatctacggcgatcactatggcaagaagaacacagaggagaagatctatctgccccctatccctgccgacgagatcagaaatcc
tgtggtgctgagggccctgtcccaggcaagaaaagtgatcaacggagtggtgcgccggtacggatctccagcccggatccacatcgagaccgccagagaagtgggcaagagcttca
aggaccggaaggagatcgagaagagacaggaggagaatcgcaaggatcgggagaaggccgccgccaagtttagggagtacttccctaactttgtgggcgagccaaagtctaagga
catcctgaagctgcgcctgtacgagcagcagcacggcaagtgtctgtatagcggcaaggagatcaatctggtgcggctgaacgagaagggctatgtggagatcgatcacgccctgcc
tttctccagaacctgggacgattcttttaacaataaggtgctggtgctgggcagcgagaaccagaataagggcaatcagacaccatacgagtatttcaatggcaaggacaactccag
ggagtggcaggagttcaaggcccgcgtggagacctctagatttcccaggagcaagaagcagcggatcctgctgcagaagttcgacgaggatggctttaaggagtgcaacctgaatg
acaccagatacgtgaaccggttcctgtgccagtttgtggccgatcacatcctgctgaccggcaagggcaagagaagggtgttcgcctctaatggccagatcacaaacctgctgagggg
attttggggactgaggaaggtgcgggcagagaatgacagacaccacgcactggatgcagtggtggtggcatgcagcaccgtggcaatgcagcagaagatcacaagattcgtgaggt
ataaggagatgaacgcctttgacggcaagaccatcgataaggagacaggcaaggtgctgcaccagaagacccacttcccccagccttgggagttctttgcccaggaagtgatgatcc
gggtgttcggcaagccagacggcaagcctgagtttgaggaggccgataccccagagaagctgaggacactgctggcagagaagctgtctagcaggccagaggcagtgcacgagtac
gtgaccccactgttcgtgtccagggcacccaatcggaagatgtctggcgcccacaaggacacactgagaagcgccaagaggtttgtgaagcacaacgagaagatctccgtgaagag
agtgtggctgaccgagatcaagctggccgatctggagaacatggtgaattacaagaacggcagggagatcgagctgtatgaggccctgaaggcaaggctggaggcctacggagga
aatgccaagcaggccttcgacccaaaggataacccctttataagaagggaggacagctggtgaaggccgtgcgggtggagaagacccaggagagcggcgtgctgctgaataaga
agaacgcctacacaatcgccgacaatggcgatatggtgagagtggacgtgttctgtaaggtggataagaagggcaagaatcagtactttatcgtgcctatctatgcctggcaggtggc
cgagaacatcctgccagacatcgattgcaagggctacagaattgacgatagctatacattctgtttttccctgcacaagtatgacctgatcgccttccagaaggatgagaagtccaagg
tggagtttgcctactatatcaattgcgactcctctaacggcaggttctacctggcctggcacgataagggcagcaaggagcagcagtttcgcatctccacccagaatctggtgctgatcc
agaagtatcaggtgaacgagctgggcaaggagatcaggccatgtcggctgaagAAGCGCCCACCCGTGCGGGAGGATAAGCGGCCCGCAGCAACCAAGAAG
GCAGGACAGGCCAAGAAGAAGAAGTAAAATAAAAGATCTTTATTTTCATTAGATCTGTGTGTTGGTTTTTTGTGTAAGCgcACGAAT***GAGGGC***
CTATTTCCCATGATTCCTTCATATTTGCATATACGATACAAGGCTGTTAGAGAGATAATTAGAATTAATTTGACTGTAAACACAAAGATATTA
GTACAAAATACGTGACGTAGAAAGTAATAATTTCTTGGGTAGTTTGCAGTTTTAAAATTATGTTTTAAAATGGACTATCATATGCTTACCGT
AACTTGAAAGTATTTCGATTTCTTGGCTTTATATATCTTGTGGAAAGGACGAAACACCNNNNNNNNNNNNNNNNNNNNNNNN**GTTGTA**
GCTCCCGAAACGTTGCTACAATAAGGCCGTCTGAAAAGATGTGCCGCAACGCTCTGCCCCTTAAAGCTTCTGCTTTAAGGGGCATCGTTTAT
TTTTTTGTTTAAACACTACCgcaggaacccctagtgatggagttggccactccctctctgcgcgctcgctcgctcactgaggccgggcgaccaaaggtcgcccgacgcccgggct
ttgcccgggcggcctcagtgagcgagcgagcgcgcagctgcctgcagg

AAV:HDR:uncleaved-Design B Plasmid to correct *eGfp* in HEK293T TLR-Multi-Cas-Variant 1 (MCV1)

Legend: ITR **sgRNA scaffold** ***eGFP* GUIDE sequence** *U6 promoter* eGFP donor *U1a promoter* NLS **hNme2Cas9** NLS short-polyA

cctgcaggcagctgcgcgctcgctcgctcactgaggccgcccgggcaaagcccgggcgtcgggcgacctttggtcgcccggcctcagtgagcgagcgagcgcgcagagagggagtggc
caactccatcactaggggttcctgcggcctctaga**GTTTAAACAAAAAAATAAACGATGCCCCTTAAAGCAGAAGCTTTAAGGGGCAGAGCGTTGCGGC**
ACATCTTTTCAGACGGCCTTATTGTAGCAACGTTTCGGGAGCTACAACCCGTATTCCACGAGGCAGGTGATc*GGTGTTTCGTCCTTTCCACAA*
GATATATAAAGCCAAGAAATCGAAATACTTTCAAGTTACGGTAAGCATATGATAGTCCATTTTAAAACATAATTTTAAAACTGCAAACTACCCA
AGAAATTATTACTTTCTACGTCACGTATTTTGTACTAATATCTTTGTGTTTACAGTCAAATTAATTCTAATTATCTCTCTAACAGCCTTGTATCGTA
*TATGCAAATATGAAGGAATCATGGGAAATAGGCCCT*CTTCCTGCCCGACCTTGACGCGCTTCTGCTTCCCGAGCtctataaaagagctcacaacccctc
actcggcgcgccagtcctccgacagactgagtcgcccggatcgatcctcgagcgccaccatggtgagcaagggcgaggagctgttcaccggggtggtgcccatcctggtcgagctggac
ggcgacgtaaacggccacaagttcagcgtgtccggcgagggcgagggcgatgccacctacggcaagctgaccctgaagttcatctgcaccaccggcaagctgcccgtgccctggccca
ccctcgtgaccaccctgacctacggcgtgcagtgcttcagccgctaccccgaccacatgaagcagcacgacttcttcaagtccgccatgcccgaaggctacgtccaggagcgcaccatct
tcttcaaggacgacggcaactacaagacccCtctaga*atggaggcggtactatgtagatgagaattcaggagcaaactgggaaaagcaactgcttccaaatatttgtgatttttac*
agtgtagttttggaaaaactcttagcctaccaattcttctaagtgttttaaaatgtgggagccagtacacatgaagttatagagtgttttaatgaggcttaaatatttaccgtaacta
*tgaaatgctacgcatatcatgctgttcaggctccgtggccacgcaactcatact*taagcagacagtggttcaaagtttttttcttccattTCAGGTGTCGTGAACACCGCCACC
ATGGTGCctaagaagaagagaaaggtggaagat**ATGGCCGCCTTCAAGCCTAACCCaatcaattacatcctgggactggacatcggaatcgcatccgtgggatgggcta**
tggtggagatcgacgaggaggagaatcctatccggctgatcgatctgggcgtgagagtgtttgagagggccgaggtgccaaagaccggcgattctctggctatggcccggagactgg
cacggagcgtgaggcgcctgacacggagaagggcacacaggctgctgagggcacgccggctgctgaagagagagggcgtgctgcaggcagcagacttcgatgagaatggcctgat
caagagcctgccaaacacccccctggcagctgagagcagccgccctggacaggaagctgacaccactggagtggtctgccgtgctgctgcacctgatcaagcaccgcggctacctgag
ccagcggaagaacgagggagagacagcagacaaggagctgggcgccctgctgaagggagtggccaacaatgcccacgccctgcagaccggcgatttcaggacacctgccgagctg
gccctgaataagtttgagaaggagtccggccacatcagaaaccagaggggcgactatagccacaccttctcccgcaaggatctgcaggccgagctgatcctgctgttcgagaagcag
aaggagtttggcaatccacacgtgagcggaggcctgaaggagggaatcgagaccctgctgatgacacagaggcctgccctgtccggcgacgcagtgcagaagatgctgggacactg
caccttcgagcctgcagagccaaaggccgccaagaacacctacacagccgagcggtttatctggctgacaaagctgaacaatctgagaatcctggagcagggatccgagaggccac
tgaccgacacagagagggccaccctgatggatgagccttaccggaagtctaagctgacatatgcccaggccagaaagctgctgggcctggaggacaccgccttctttaagggcctga
gatacggcaaggataatgccgaggcctccacactgatggagatgaaggcctatcacgccatctctcgcgccctggagaaggagggcctgaaggacaagaagtccccctgaacctg
agctccgagctgcaggatgagatcggcaccgccttctctctgtttaagaccgacgaggatatcacaggccgcctgaaggacagggtgcagcctgagatcctggaggccctgctgaagc
acatctctttcgataagtttgtgcagatcagcctgaaggccctgagaaggatcgtgccactgatggagcagggcaagcggtacgacgaggcctgcgccgagatctacggcgatcacta
tggcaagaagaacacagaggagaagatctatctgccccctatccctgccgacgagatcagaaatcctgtggtgctgagggccctgtcccaggcaagaaaagtgatcaacggagtggt
gcgccggtacggatctccagcccggatccacatcgagaccgccagagaagtgggcaagagcttcaaggaccggaaggagatcgagaagagacaggaggagaatcgcaaggatcg
ggagaaggccgccgccaagtttagggagtacttccctaactttgtgggcgagccaaagtctaaggacatcctgaagctgcgcctgtacgagcagcagcacggcaagtgtctgtatagc
ggcaaggagatcaatctggtgcggctgaacgagaagggctatgtggagatcgatcacgccctgcctttctccagaacctgggacgattcttttaacaataaggtgctggtgctgggca
gcgagaaccagaataagggcaatcagacaccatacgagtatttcaatggcaaggacaactccagggagtggcaggagttcaaggcccgcgtggagacctctagatttcccaggagc
aagaagcagcggatcctgctgcagaagttcgacgaggatggctttaaggagtgcaacctgaatgacaccagatacgtgaaccggttcctgtgccagtttgtggccgatcacatcctgc
tgaccggcaagggcaagagaagggtgttcgcctctaatggccagatcacaaacctgctgaggggattttggggactgaggaaggtgcgggcagagaatgacagacaccacgcactg
gatgcagtggtggtggcatgcagcaccgtggcaatgcagcagaagatcacaagattcgtgaggtataaggagatgaacgcctttgacggcaagaccatcgataaggagacaggca
aggtgctgcaccagaagacccacttccccccagccttgggagttctttgcccaggaagtgatgatccgggtgttcggcaagccagacggcaagcctgagtttgaggaggccgatacccc
agagaagctgaggacactgctggcagagaagctgtctagcaggccagaggcagtgcacgagtacgtgaccccactgttcgtgtccagggcacccaatcggaagatgtctggcgccca
caaggacacactgagaagcgccaagaggtttgtgaagcacaacgagaagatctccgtgaagagagtgtggctgaccgagatcaagctggccgatctggagaacatggtgaattac
aagaacggcagggagatcgagctgtatgaggccctgaaggcaaggctggaggcctacggaggaaatgccaagcaggccttcgacccaaaggataaccccttttataagaagggag
gacagctggtgaaggccgtgcgggtggagaagacccaggagagcggcgtgctgctgaataagaagaacgcctacacaatcgccgacaatggcgatatggtgagagtggacgtgttc
tgtaaggtggataagaagggcaagaatcagtactttatcgtgcctatctatgcctggcaggtggccgagaacatcctgccagacatcgattgcaagggctacagaatcgacgatagct
atacattctgtttttccctgcacaagtatgacctgatcgccttccagaaggatgagaagtccaaggtggagtttgcctactatatcaattgcgactcctctaacggcaggttctacctggc
ctggcacgataagggcagcaaggagcagcagtttcgcatctccacccagaatctggtgctgatccagaagtatcaggtgaacgagctgggcaaggagatcaggccatgtcggctgaa
gAAGCGCCCACCCGTGCGGGAGGATAAGCGGCCCGCAGCAACCAAGAAGGCAGGACAGGCCAAGAAGAAGAAGTAAAATAAAAGATCTTT
ATTTTCATTAGATCTGTGTGTTGGTTTTTTGTGTAAGCgcggccgcaggaacccctagtgatggagttggccactccctctctgcgcgctcgctcgctcactgaggccggg
cgaccaaaggtcgcccgacgcccgggctttgcccgggcggcctcagtgagcgagcgagcgcgcagctgcctgcagg

AAV:HDR:cleaved-Design D Plasmid to correct *eGfp* in HEK293T TLR-Multi-Cas-Variant 1 (MCV1)

Legend: ITR **sgRNA scaffold** ***eGFP* GUIDE sequence** *U6 promoter* *eGFP* Target_site-1 eGFP donor
eGFP Target_site-2 *U1a promoter* NLS **hNme2Cas9** NLS short-polyA

cctgcaggcagctgcgcgctcgctcgctcactgaggccgcccgggcaaagcccgggcgtcgggcgacctttggtcgcccggcctcagtgagcgagcgagcgcgcagagagggagtggc
caactccatcactaggggttcctgcggcctctaga**GTTTAAACAAAAAAATAAACGATGCCCCTTAAAGCAGAAGCTTTAAGGGGCAGAGCGTTGCGGC**
ACATCTTTTCAGACGGCCTTATTGTAGCAACGTTTCGGGAGCTACAACCCGTATTCCACGAGGCAGGTGATc*GGTGTTTCGTCCTTTCCACAA*
GATATATAAAGCCAAGAAATCGAAATACTTTCAAGTTACGGTAAGCATATGATAGTCCATTTTAAAACATAATTTTAAAACTGCAAACTACCCA
AGAAATTATTACTTTCTACGTCACGTATTTTGTACTAATATCTTTGTGTTTACAGTCAAATTAATTCTAATTATCTCTCTAACAGCCTTGTATCGTA
*TATGCAAATATGAAGGAATCATGGGAAATAGGCCCT*CTTCCTGCCCGACCTTGACGAATCACCTGCCTCGTGGAATACGGTAAACCTgcgcttct
gcttcccgagctctataaaagagctcacaacccctcactcggcgcgccagtcctccgacagactgagtcgcccggatcgatcctcgagcgccaccatggtgagcaagggcgaggagctg
ttcaccggggtggtgcccatcctggtcgagctggacggcgacgtaaacggccacaagttcagcgtgtccggcgagggcgagggcgatgccacctacggcaagctgaccctgaagttcat
ctgcaccaccggcaagctgcccgtgccctggcccaccctcgtgaccaccctgacctacggcgtgcagtgcttcagccgctaccccgaccacatgaagcagcacgacttcttcaagtccgc
catgcccgaaggctacgtccaggagcgcaccatcttcttcaaggacgacggcaactacaagacccAGGTTTACCGTATTCCACGAGGCAGGTGATTCtctaga*atggag*
gcggtactatgtagatgagaattcaggagcaaactgggaaaagcaactgcttccaaatatttgtgatttttacagtgtagttttggaaaaactcttagcctaccaattcttctaagt
gttttaaaatgtgggagccagtacacatgaagttatagagtgttttaatgaggcttaaatatttaccgtaactatgaaatgctacgcatatcatgctgttcaggctccgtggccacg
*caactcatact*taagcagacagtggttcaaagtttttttcttccattTCAGGTGTCGTGAACACCGCCACCATGGTGCctaagaagaagagaaaggtggaagat**ATGGC**
CGCCTTCAAGCCTAACCCaatcaattacatcctgggactggacatcggaatcgcatccgtgggatgggctatggtggagatcgacgaggaggagaatcctatccggctgatcg
atctgggcgtgagagtgtttgagagggccgaggtgccaaagaccggcgattctctggctatggcccggagactggcacggagcgtgaggcgcctgacacggagaagggcacacagg
ctgctgagggcacgccggctgctgaagagagagggcgtgctgcaggcagcagacttcgatgagaatggcctgatcaagagcctgccaaacacccctggcagctgagagcagccgc
cctggacaggaagctgacaccactggagtggtctgccgtgctgctgcacctgatcaagcaccgcggctacctgagccagcggaagaacgagggagagacagcagacaaggagctg
ggcgccctgctgaagggagtggccaacaatgcccacgccctgcagaccggcgatttcaggacacctgccgagctggccctgaataagtttgagaaggagtccggccacatcagaaac
cagaggggcgactatagccacaccttctcccgcaaggatctgcaggccgagctgatcctgctgttcgagaagcagaaggagtttggcaatccacacgtgagcggaggcctgaaggag
ggaatcgagaccctgctgatgacacagaggcctgccctgtccggcgacgcagtgcagaagatgctgggacactgcaccttcgagcctgcagagccaaaggccgccaagaacaccta
cacagccgagcggtttatctggctgacaaagctgaacaatctgagaatcctggagcagggatccgagaggccactgaccgacacagagagggccaccctgatggatgagccttacc
ggaagtctaagctgacatatgcccaggccagaaagctgctgggcctggaggacaccgccttctttaagggcctgagatacggcaaggataatgccgaggcctccacactgatggaga
tgaaggcctatcacgccatctctcgcgccctggagaaggagggcctgaaggacaagaagtccccccctgaacctgagctccgagctgcaggatgagatcggcaccgccttctctctgttt
aagaccgacgaggatatcacaggccgcctgaaggacagggtgcagcctgagatcctggaggccctgctgaagcacatctctttcgataagtttgtgcagatcagcctgaaggccctg
agaaggatcgtgccactgatggagcagggcaagcggtacgacgaggcctgcgccgagatctacggcgatcactatggcaagaagaacacagaggagaagatctatctgccccctat
ccctgccgacgagatcagaaatcctgtggtgctgagggccctgtcccaggcaagaaaagtgatcaacggagtggtgcgccggtacggatctccagcccggatccacatcgagaccgc
cagagaagtgggcaagagcttcaaggaccggaaggagatcgagaagagacaggaggagaatcgcaaggatcgggagaaggccgccgccaagtttagggagtacttccctaacttt
gtgggcgagccaaagtctaaggacatcctgaagctgcgcctgtacgagcagcagcacggcaagtgtctgtatagcggcaaggagatcaatctggtgcggctgaacgagaagggcta
tgtggagatcgatcacgccctgcctttctccagaacctgggacgattcttttaacaataaggtgctggtgctgggcagcgagaaccagaataagggcaatcagacaccatacgagtat
ttcaatggcaaggacaactccagggagtggcaggagttcaaggcccgcgtggagacctctagatttcccaggagcaagaagcagcggatcctgctgcagaagttcgacgaggatgg
ctttaaggagtgcaacctgaatgacaccagatacgtgaaccggttcctgtgccagtttgtggccgatcacatcctgctgaccggcaagggcaagagaagggtgttcgcctctaatggcc
agatcacaaacctgctgaggggattttggggactgaggaaggtgcgggcagagaatgacagacaccacgcactggatgcagtggtggtggcatgcagcaccgtggcaatgcagcag
aagatcacaagattcgtgaggtataaggagatgaacgcctttgacggcaagaccatcgataaggagacaggcaaggtgctgcaccagaagacccacttcccccagccttgggagtt
ctttgcccaggaagtgatgatccgggtgttcggcaagccagacggcaagcctgagtttgaggaggccgataccccagagaagctgaggacactgctggcagagaagctgtctagcag
gccagaggcagtgcacgagtacgtgaccccactgttcgtgtccagggcacccaatcggaagatgtctggcgcccacaaggacacactgagaagcgccaagaggtttgtgaagcaca
acgagaagatctccgtgaagagagtgtggctgaccgagatcaagctggccgatctggagaacatggtgaattacaagaacggcagggagatcgagctgtatgaggccctgaaggc
aaggctggaggcctacggaggaaatgccaagcaggccttcgacccaaaggataacccctttataagaagggaggacagctggtgaaggccgtgcgggtggagaagacccaggag
agcggcgtgctgctgaataagaagaacgcctacacaatcgccgacaatggcgatatggtgagagtggacgtgttctgtaaggtggataagaagggcaagaatcagtactttatcgtg
cctatctatgcctggcaggtggccgagaacatcctgccagacatcgattgcaagggctacagaatcgacgatagctatacattctgtttttccctgcacaagtatgacctgatcgccttc
cagaaggatgagaagtccaaggtggagtttgcctactatatcaattgcgactcctctaacggcaggttctacctggcctggcacgataagggcagcaaggagcagcagtttcgcatct
ccacccagaatctggtgctgatccagaagtatcaggtgaacgagctgggcaaggagatcaggccatgtcggctgaagAAGCGCCCACCCGTGCGGGAGGATAAGCGG
CCCGCAGCAACCAAGAAGGCAGGACAGGCCAAGAAGAAGAAGTAAAATAAAAGATCTTTATTTTCATTAGATCTGTGTGTTGGTTTTTTGTGT
AAGCgcggccgcaggaacccctagtgatggagttggccactccctctctgcgcgctcgctcgctcactgaggccgggcgaccaaaggtcgcccgacgcccgggctttgcccgggcggc
ctcagtgagcgagcgagcgcgcagctgcctgcagg

Figure exemplifying the gating strategy of flow cytometry:

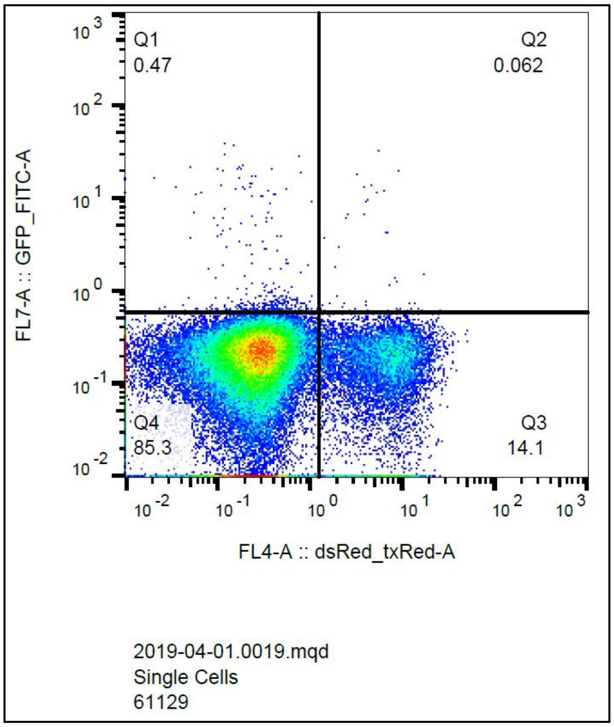

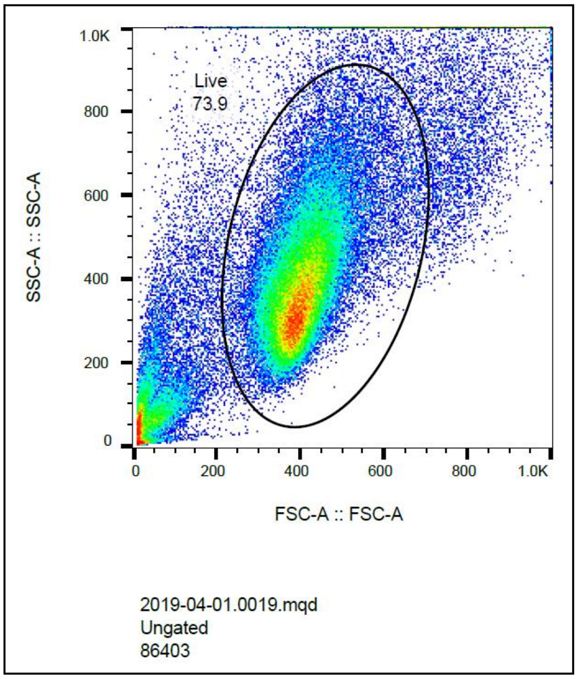

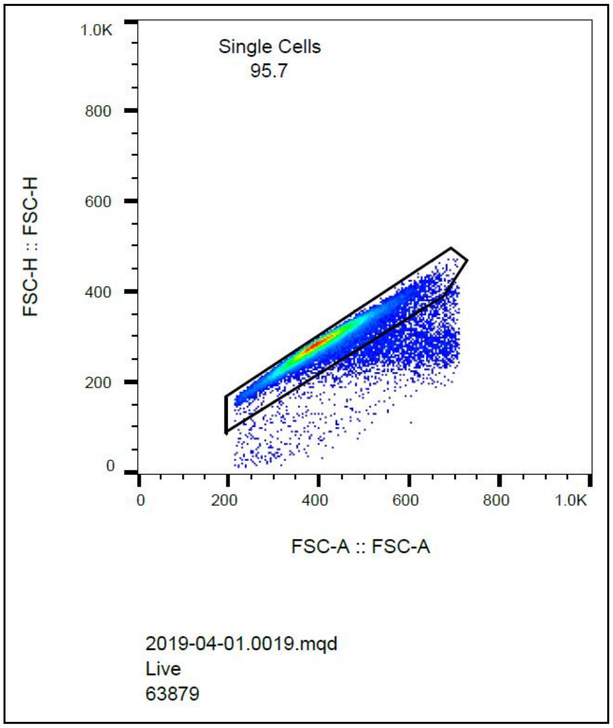

Supplementary Tables

Primers for amplicon seq, qPCR, TIDE, SMRT & UDiTaS

Gene	Application	Primer Name	Sequence (5'-3')	Notes
Hpd	SMRT seq	FWD	**gcagtcgaacatgtagctgactcaggtcac***NNWNNBVH*AACGCAACAACCCTCTACCC	Gene specific sequence is underlined, UMI is italicized, and PacBio adapter sequences are in bold
		REV	**tggatcacttgtgcaagcatcacatcgtag**GCCCAACACCTCTAGCTACC	
Hpd gRNA-I site	UDiTaS seq	FWD (Hpd_2.22_FWD)	**GTGACTGGAGTTCAGACGTGTGCTCTTCCGATCT**GGAATTTCCCAATTCTGATCCT	Gene specific sequence is underlined, and Illumina adapter sequences are in bold
		REV (Hpd_2.22_REV)	**GTGACTGGAGTTCAGACGTGTGCTCTTCCGATCT**GGAGGAACGAGGAGGATG	
Hpd gRNA-II site	UDiTaS seq	FWD (Hpd_4.10_FWD)	**GTGACTGGAGTTCAGACGTGTGCTCTTCCGATCT**CAAGATGGGCTTTGAACCTC	Gene specific sequence is underlined, and Illumina adapter sequences are in bold
		REV (Hpd_4.10_REV)	**GTGACTGGAGTTCAGACGTGTGCTCTTCCGATCT**AGCCATACATCTTGGAACCAG	
Hpd	UDiTaS seq	Hpd_2.22_nest_FWD	TGGTCACCCATACTGTTCTCACG	Nested PCR for making the UDiTaS library
TS6 *LINC01588*	TIDE	FWD	AGAGGAGCCTTCTGACTGCTGCAGA	
		REV	ATGACAGACACAACCAGAGGGCA	
TS16 *LINC01588*	TIDE	FWD	AGAGGAGCCTTCTGACTGCTGCAGA	
		REV	ATGACAGACACAACCAGAGGGCA	
TS47 *VEGFA*	TIDE	FWD	GTACATGAAGCAACTCCAGTCCCA	
		REV	ATCAAATTCCAGCACCGAGCGC	
CYBB	TIDE	FWD	TAGAGAACTGGGTAGTGTG	
		REV	CCAATATTGCATGGGATGG	
Pcsk9	TIDE	FWD	GGCTCCCGTTCTCTCTCTCT	
		REV	CGCTAAATCGAGGCCTACAG	
Fah	Amplicon seq	FWD	**CTACACGACGCTCTTCCGATCT**CCCTAGTCCCTGGTTGAAC	Gene specific sequence is underlined, and illumina adapter sequences are in bold
		REV	**agacgtgtgctcttccgatct**GTGGAGTGTGTAGGGCTTG	
Fah	qRT-PCR	FWD	cggacttctactcttctcgg	Amplify Fah cDNA exons 5-9. FWD primer is complementary to exon 5 whereas REV is complementary to exon 9
		REV	ggagattgtggttccaaagc	
Fah	qRT-PCR	FWD	agagccaatccccatttcca	Amplify Fah cDNA exons 8 & 9. FWD primer is complementary to exon 8 whereas REV is complementary to exon 9
		REV	ctgaacataatgccaacattgg	
U6 <> FahDonor	qPCR of AAV	FWD	AAGGAATCATGGGAAATAGGC	
		REV	CTGGGTCACTGTGGCTTACA	
FahDonor <> U1a	qPCR of AAV	FWD	AAGTGGAAGGATGGAGCTGA	
		REV	CTTTTCCCAGTTTGCTCCTG	

Nme2Cas9 mRNA	qRT-PCR	FWD	accgacgaggatatcacagg	
		REV	tcaggctgatctgcacaaac	
Fah gRNA expression	qRT-PCR	FWD	CCTCATGAACGACTGGAGCaGTG	
		REV	GGGGCAGAGCGTTGCGGC	
Idua	TIDE	FWD	CCTGGCACATCCTGTATTGA	
		REV	GCATACGTCGGAACTGCTCT	
Idua	Amplicon seq	FWD	**ctacacgacgctcttccgatct**gataccgtcgagggacctaa	Gene specific sequence is underlined, and illumina adapter sequences are in bold
		REV	**agacgtgtgctcttccgatct**AGAACCCCACTCACCCAAG	
Idua	qRT-PCR	FWD	CCTCCTGGCTTGGATCTTGTCTAC	Amplify Idua cDNA exons 10 & 11. FWD primer is complementary to exons 10 & 11, whereas REV is complementary to exon 11
		REV	CTCCACCATGCGCATACGT	
Idua gRNA expression	qRT-PCR	FWD	CGTTGCGGCACATCTTT	
		REV	TTGAGACCTCTGCCTAGAGT	
U6 <> IduaDonor	qPCR of AAV	FWD	AAGGAATCATGGGAAATAGGC	
		REV	AAACTCACACAGCAGCTGGA	
IduaDonor <> U1a	qPCR of AAV	FWD	ACACCCCAACCACAGTATCC	
		REV	ttcccagtttgctcctgaat	
Amplicon seq of rAAV:HDR:cleaved re-ligation after donor excision	Amplicon seq	FWD	**CTACACGACGCTCTTCCGATCT***NNWNNBVH* GCAAATATGAAGGAATCATGGGAA	Gene specific sequence is underlined, UMI is italicized, and Ilumina adapter sequences are in bold
		REV	**agacgtgtgctcttccgatct**gtttgctcctgaattctcatctaca	
		Universal FWD	CTACACGACGCTCTTCCGA	

Off-target primers

Gene [On/off #]	Forward primer name	Forward primer [5'-3']	Reverse primer name	Reverse primer [5'-3']
Hpd gRNA-I ON	DS_HpdgRNAI-ON_F	ctacacgacgctcttccgatct**TTTCCTTCCATGACCCCTGG**	DS_HpdgRNAI-ON_R	agacgtgtgctcttccgatct**GAAATGGAGGAACCGGCCTC**
Hpd gRNA-I OT1 (Rbm20)	DS_HpdgRNAI-OT1_F	ctacacgacgctcttccgatct**GCCTCTGAGCTTGTATTTCCTCA**	DS_HpdgRNAI-OT1_R	agacgtgtgctcttccgatct**AGAGGGAACTTAGCGCCCATT**
Hpd gRNA-I OT2 (Pmp2)	DS_HpdgRNAI-OT2_F	ctacacgacgctcttccgatct**AGGTGCCTAGGAATTTGTTGCTC**	DS_HpdgRNAI-OT2_R	agacgtgtgctcttccgatct**ACTTAAGAGCCATTCATCACTCAGC**
Hpd gRNA-I OT3 (unannot)	DS_HpdgRNAI-OT3_F	ctacacgacgctcttccgatct**ACTGGTCAATTTTGACAGCTTGGA**	DS_HpdgRNAI-OT3_R	agacgtgtgctcttccgatct**CCGTGGGGAGCAGGAATTTTAG**
Hpd gRNA-I OT4 (Btbd16)	DS_HpdgRNAI-OT4_F	ctacacgacgctcttccgatct**GTTATCTGCCTCCTTCTGGCTTAC**	DS_HpdgRNAI-OT4_R	agacgtgtgctcttccgatct**CTAGGTCACTCTAATGTGTCCCCA**
Hpd gRNA-II ON	DS_HpdgRNAII-ON_F	ctacacgacgctcttccgatct**CCAGGCTGCTTCCTTCTACT**	DS_HpdgRNAII-ON_R	agacgtgtgctcttccgatct**GGAGGGACATGGGTCAGTT**
Hpd gRNA-II OT1 (unannot)	DS_HpdgRNAII-OT1_F	ctacacgacgctcttccgatct**CATTCTACAACTGTCTGGACCTTTCTG**	DS_HpdgRNAII-OT1_R	agacgtgtgctcttccgatct**ACAGAGATCACAGGGAAGTATAGTCAC**
Hpd gRNA-II OT2 (Rabgap1l)	DS_HpdgRNAII-OT2_F	ctacacgacgctcttccgatct**ACATTCTGGAGAGGGTGACCTG**	DS_HpdgRNAII-OT2_R	agacgtgtgctcttccgatct**GCCCTGAAGTTGTTAAGAGGTGAG**
Hpd gRNA-II OT3 (Maml2)	DS_HpdgRNAII-OT3_F	ctacacgacgctcttccgatct**GAAAACTCGTTATCTCTCTGTGAAGATTTCTGG**	DS_HpdgRNAII-OT3_R	agacgtgtgctcttccgatct**GGATGCTGGCATCAATTCAATGAAAAC**
Hpd gRNA-II OT4 (Btbd35f20)	DS_HpdgRNAII-OT4_F	ctacacgacgctcttccgatct**GCCTGGGCTAAATATCCTGTCTG**	DS_HpdgRNAII-OT4_R	agacgtgtgctcttccgatct**GGAGTCCAGGAGTGAGTGTTCTAAG**
Fah OT1 (Chka)	DS_Fah-OT1_F	ctacacgacgctcttccgatct**TGGTTCTGGAGAGTGTTATGTTTGC**	DS_Fah-OT1_R	agacgtgtgctcttccgatct**ATCAAAAGGAATGTGCTCCCAGAGA**
Fah OT2 (Gli3)	DS_Fah-OT2_F	ctacacgacgctcttccgatct**CTCCCTCTGTCATCCGTCATTG**	DS_Fah-OT2_R	agacgtgtgctcttccgatct**AACAGGCTTACACTAGGCAGAGA**
Fah OT3 (Acvr1)	DS_Fah-OT3_F	ctacacgacgctcttccgatct**GGCTCTGGGTTGACTTGGAATTTAT**	DS_Fah-OT3_R	agacgtgtgctcttccgatct**CAAGAAAACAGGGCTGAAACTGG**
Fah OT4 (unannot)	DS_Fah-OT4_F	ctacacgacgctcttccgatct**GTGGTACTCAACCTGTGGGT**	DS_Fah-OT4_R	agacgtgtgctcttccgatct**TCCCTAGCTGCTGCTCCA**
Fah OT5 (Saxo1)	DS_Fah-OT5_F	ctacacgacgctcttccgatct**TCTGCAAAGGATTTTACAGCTACAT**	DS_Fah-OT5_R	agacgtgtgctcttccgatct**CTTGTAGGTAGCAGAGCTCAGAA**
Idua OT1 (unannot)	DS_Idua-OT1_F	ctacacgacgctcttccgatct**GAATGCCTTAAAAGACTGGTCCCT**	DS_Idua-OT1_R	agacgtgtgctcttccgatct**CCATGCTCCTGAATATGTAAGTGTGAATC**
Idua OT2 (unannot)	DS_Idua-OT2_F	ctacacgacgctcttccgatct**CATGCCCAAGAGAGACACCA**	DS_Idua-OT2_R	agacgtgtgctcttccgatct**CTGGAAACATCATCTATCACAGGGAC**
Idua OT3 (Amph)	DS_Idua-OT3_F	ctacacgacgctcttccgatct**GGAAAGAACACATCGGCCTTTTG**	DS_Idua-OT3_R	agacgtgtgctcttccgatct**CCAGGCATTGGACCACCTTTC**
Idua OT4 (Acot12)	DS_Idua-OT4_F	ctacacgacgctcttccgatct**GCATCATCACAAAAGCAGAAGCTG**	DS_Idua-OT4_R	agacgtgtgctcttccgatct**TCACTCGTTGCAAATCAAGGCA**
Idua OT5 (unannot)	DS_Idua-OT5_F	ctacacgacgctcttccgatct**GGGAAGGCAGATTGATAGTCGTG**	DS_Idua-OT5_R	agacgtgtgctcttccgatct**TTTCCCTTCAGGAAACAGTGTGG**

Tagmentation primers

Oligo Name	5p-Index Name	5p-Index Sequence	5p-Primer Sequence
UDiTaS adapter top i501	i501	TATAGCCT	AATGATACGGCGACCACCGAGATCTACAC**TATAGCCT**NNWNNWNNNTCGTCGGCAGCGTCAGATGTGTATAAGAGACAG
UDiTaS adapter top i502	i502	ATAGAGGC	AATGATACGGCGACCACCGAGATCTACAC**ATAGAGGC**NNWNNWNNNTCGTCGGCAGCGTCAGATGTGTATAAGAGACAG
UDiTaS adapter top i503	i503	CCTATCCT	AATGATACGGCGACCACCGAGATCTACAC**CCTATCCT**NNWNNWNNNTCGTCGGCAGCGTCAGATGTGTATAAGAGACAG
UDiTaS adapter top i504	i504	GGCTCTGA	AATGATACGGCGACCACCGAGATCTACAC**GGCTCTGA**NNWNNWNNNTCGTCGGCAGCGTCAGATGTGTATAAGAGACAG
UDiTaS adapter top i505	i505	AGGCGAAG	AATGATACGGCGACCACCGAGATCTACAC**AGGCGAAG**NNWNNWNNNTCGTCGGCAGCGTCAGATGTGTATAAGAGACAG
UDiTaS adapter top i506	i506	TAATCTTA	AATGATACGGCGACCACCGAGATCTACAC**TAATCTTA**NNWNNWNNNTCGTCGGCAGCGTCAGATGTGTATAAGAGACAG
UDiTaS adapter top i507	i507	CAGGACGT	AATGATACGGCGACCACCGAGATCTACAC**CAGGACGT**NNWNNWNNNTCGTCGGCAGCGTCAGATGTGTATAAGAGACAG
UDiTaS adapter top i508	i508	GTACTGAC	AATGATACGGCGACCACCGAGATCTACAC**GTACTGAC**NNWNNWNNNTCGTCGGCAGCGTCAGATGTGTATAAGAGACAG
UDiTaS adapter top i509	i509	GACGACCT	AATGATACGGCGACCACCGAGATCTACAC**GACGACCT**NNWNNWNNNTCGTCGGCAGCGTCAGATGTGTATAAGAGACAG
UDiTaS adapter top i510	i510	TAATCGGC	AATGATACGGCGACCACCGAGATCTACAC**TAATCGGC**NNWNNWNNNTCGTCGGCAGCGTCAGATGTGTATAAGAGACAG
UDiTaS adapter top i511	i511	TACAGCCT	AATGATACGGCGACCACCGAGATCTACAC**TACAGCCT**NNWNNWNNNTCGTCGGCAGCGTCAGATGTGTATAAGAGACAG
UDiTaS adapter top i512	i512	TATAATGA	AATGATACGGCGACCACCGAGATCTACAC**TATAATGA**NNWNNWNNNTCGTCGGCAGCGTCAGATGTGTATAAGAGACAG
UDiTaS adapter top i513	i513	CAGGCGAG	AATGATACGGCGACCACCGAGATCTACAC**CAGGCGAG**NNWNNWNNNTCGTCGGCAGCGTCAGATGTGTATAAGAGACAG
UDiTaS adapter top i514	i514	CATGGCTA	AATGATACGGCGACCACCGAGATCTACAC**CATGGCTA**NNWNNWNNNTCGTCGGCAGCGTCAGATGTGTATAAGAGACAG
UDiTaS adapter top i515	i515	ATGAGCGT	AATGATACGGCGACCACCGAGATCTACAC**ATGAGCGT**NNWNNWNNNTCGTCGGCAGCGTCAGATGTGTATAAGAGACAG
UDiTaS adapter top i516	i516	GGTAGCAC	AATGATACGGCGACCACCGAGATCTACAC**GGTAGCAC**NNWNNWNNNTCGTCGGCAGCGTCAGATGTGTATAAGAGACAG

Nme2Cas9 Target sites

**Gene**	**Sequence**
TLR-MCV1 Locus	AATCACCTGCCTCGTGGAATACGG<u>TAAA**CC**</u>
TS6 *LINC01588*	GCCTCCCTGCAGGGCTGCTCCC<u>CAGC**CC**</u>
TS16 *LINC01588*	GGAGTCGCCAGAGGCCGGTGGTGG<u>ATTT**CC**</u>
TS47 *VEGFA*	GTGTGTCCCTCTCCCCACCCGTCC<u>CTGT**CC**</u>
CYBB	GAGGAAGGGAACATATTACTATTG<u>CTTT**CC**</u>
Pcsk9	GGCCTGGCTGATGAGGCCGCACAT<u>GTGG**CC**</u>
Hpd gRNA-I	ACCTCCGAGAATGGGACTCACCCT<u>GCTC**CC**</u>
Hpd gRNA-II	CATCAAGCAAGGGAAAGTGAGTAC<u>ACAT**CC**</u>
Fah	GTCCTCATGAACGACTGGAGCaGT<u>AATG**CC**</u>
Idua	CTTTGAGACCTCTGCCCAGAGTTG<u>TTCT**CC**</u>

Self-inactivation AAV NGS (*Fah*)

Mouse	Cohort	AAV amplicons with donor [reads by NGS]	AAV amplicons without the donor [reads by NGS]	AAV amplicons with donor inverted in the backbone [reads by NGS]
EM2992	rAAV:HDR:Uncleaved [Pre-NTBC withdrawal mice]	8538	0	0
EM3143		9242	0	0
EM3144		8888	0	0
EM2989	rAAV:HDR:Uncleaved [Post-NTBC withdrawal mice]	8229	0	0
EM2990		6772	0	0
EM2991		8197	0	0
EM2997		7356	0	0
EM2998		5701	0	0
EM2999		8474	0	0
EM3000		8490	0	0
EM2996	rAAV:HDR:Cleaved [Pre-NTBC withdrawal mice]	13	15944 [reads with indels 37%]	5
EM3145		20	16025 (reads with indels 38%]	13
EM3146		18	15947 [reads with indels 24.1%]	10
EM2993	rAAV:HDR:Cleaved [Post-NTBC withdrawal mice]	27	13378 [reads with indels 62.8%]	20
EM2994		10	14490 [reads with indels 64.9%]	4
EM2995		8	15174 [reads with indels 67.3%]	1
EM2927		3	16382 [reads with indels 87.6%]	2
EM2928		4	15035 [reads with indels 83.8%]	1
EM2929		8	15091 [reads with indels 65.4%]	1
EM2930		3	13829 [reads with indels 83.1%]	1

Self-inactivation AAV NGS(*Idua*)

Mouse	Cohort	AAV amplicons with donor [reads by NGS]	AAV amplicons without the donor [reads by NGS]	AAV amplicons with donor inverted in the backbone [reads by NGS]
8836	rAAV:HDR:uncleaved	214	0	0
8838		181	0	0
8848		266	0	0
8849		250	0	0
8835	rAAV:HDR:cleaved	1	6759 [reads with indels 99.4%]	1
8837		0	6942 [reads with indels 98.1%]	0
8839		0	6294 [reads with indels 98.9]	0
8850		4	8844 [reads with indels 99.2%]	4
8851		1	5436 [reads with indels 99.6%]	1

CRISPRseek Analysis

name	# of OT sites	gRNAPlusPAM	OffTargetSequence	inExon	inIntron	gene	score	n.mismatch	mismatch.distance2PAM	alignment	isCanonicalPAM	forViewInUCSC	strand	chrom	chromStart	chromEnd
Hpd gRNA-I	On target site	**ACCTCCGAGAATGGGACTCACCCTNNNNCC**	**ACCTCCGAGAATGGGACTCACCCTGCTCCC**	TRUE		***Hpd***	**100**	**0**		....	**1**	**chr5:123182003-123182032**	**+**	**chr5**	**123182003**	**123182032**
	1	ACCTCCGAGAATGGGACTCACCCTNNNNCC	TCCTACCTGAAGGAGACTCACCCTCATCCC		TRUE	*Rbm20*	0.6	6	24,20,18,17,13,11	T...A.CT...G.A..........	1	chr19:53708347-53708376	-	chr19	53708347	53708376
	2	ACCTCCGAGAATGGGACTCACCCTNNNNCC	AACCCAAAGCATGTGACTCACCCTGAGACC		TRUE	*Pmp2*	0.3	6	23,21,19,18,15,11	.A.C.AA..C...T..........	1	chr3:10183963-10183992	+	chr3	10183963	10183992
	3	ACCTCCGAGAATGGGACTCACCCTNNNNCC	GCCTTCGTTAGTAGGACTCACCCTTTAGCC				0.3	6	24,20,17,16,14,12	G...T..TT.G.A...........	1	chr4:142419033-142419062	+	chr4	142419033	142419062
	4	ACCTCCGAGAATGGGACTCACCCTNNNNCC	GGCTTTGAGGTTGGGACTCACCCTGGTACC		TRUE	*Btbd16*	0.3	6	24,23,20,19,15,14	GG..TT...GT.............	1	chr7:130823801-130823830	+	chr7	130823801	130823830

name	# of OT sites	gRNAPlusPAM	OffTargetSequence	inExon	inIntron	gene	score	n.mismatch	mismatch.distance2PAM	alignment	isCanonicalPAM	forViewInUCSC	strand	chrom	chromStart	chromEnd
Hpd gRNA-II	On target site	**CATCAAGCAAGGGAAAGTGAGTACNNNNCC**	**CATCAAGCAAGGGAAAGTGAGTACACATCC**	TRUE		**Hpd**	**100**	**0**		....	**1**	**chr5:123181409-123181438**	**-**	**chr5**	**123181409**	**123181438**
	1	CATCAAGCAAGGGAAAGTGAGTACNNNNCC	TTACAGGAAAGGGAAAGTGAGTACACACCC				0.9	5	24,23,22,19,17	TTA..G.A................	1	chr18:23160946-23160975	-	chr18	23160946	23160975
	2	CATCAAGCAAGGGAAAGTGAGTACNNNNCC	AAGTGAGCAAAGGCAAGTGAGTACCCATCC		TRUE	*Rabgap1l*	0.4	6	24,22,21,20,14,11	A.GTG.....A..C..........	1	chr1:160367957-160367986	-	chr1	160367957	160367986
	3	CATCAAGCAAGGGAAAGTGAGTACNNNNCC	CACCAAGCACATGCAAGTGAGTACTAACCC		TRUE	*Maml2*	0.3	5	22,15,14,13,11	..C......CAT.C..........	1	chr9:13493623-13493652	-	chr9	13493623	13493652
	4	CATCAAGCAAGGGAAAGTGAGTACNNNNCC	CATGCTGCAAAGGATAGTGAGTACTGGGCC		TRUE	*Btbd35f20*	0.3	5	21,20,19,14,10	...GCT....A...T.........	1	chrX:11658687-11658716	-	chrX	11658687	11658716

name	# of OT sites	gRNAPlusPAM	OffTargetSequence	inExon	inIntron	gene	score	n.mismatch	mismatch.distance2PAM	alignment	isCanonicalPAM	forViewInUCSC	strand	chrom	chromStart	chromEnd
Fah	On target site	**GTCCTCATGAACGACTGGAGCAGTNNNNCC**	**GTCCTCATGAACGACTGGAGCaGTAATGCC**	TRUE		**Fah_tyrosinemia**	**19.6**	**1**	**3**	.....................**G**..	**1**	**chr7:84595450-84595479**	**-**	**chr7**	**84595450**	**84595479**
	1	GTCCTCATGAACGACTGGAGCAGTNNNNCC	TTCCCCAAGGCCGACTGGAGCAGTTTATCC	TRUE		*Chka*	0.4	5	24,20,17,15,14	T...C..A.GC.............	1	chr19:3884554-3884583	+	chr19	3884554	3884583
	2	GTCCTCATGAACGACTGGAGCAGTNNNNCC	GGGCTCAGGAACGCCTAGAGCAGTGATTCC		TRUE	*Gli3*	0.3	5	23,22,17,11,8	.GG....G.....C..A.......	1	chr13:15542329-15542358	-	chr13	15542329	15542358
	3	GTCCTCATGAACGACTGGAGCAGTNNNNCC	ATAGCCATGAACCACAGGAGCAGTCTGCCC		TRUE	*Acvr1*	0.2	6	24,22,21,20,12,9	A.AGC.......C..A........	1	chr2:58552372-58552401	-	chr2	58552372	58552401
	4	GTCCTCATGAACGACTGGAGCAGTNNNNCC	GTCGTTATAAACCTGTGGAGCAGTGCTTCC				0.2	6	21,19,16,12,11,10	...G.T..A...CTG.........	1	chr1:92481526-92481555	-	chr1	92481526	92481555
	5	GTCCTCATGAACGACTGGAGCAGTNNNNCC	TTGCTCATGAACAGCTGGAGCAATGACACC		TRUE	*Saxo1*	0.2	5	24,22,12,11,2	T.G.........AG........A.	1	chr4:86556873-86556902	+	chr4	86556873	86556902

name	# of OT sites	gRNAPlusPAM	OffTargetSequence	inExon	inIntron	gene	score	n.mismatch	mismatch.distance2PAM	alignment	isCanonicalPAM	forViewInUCSC	strand	chrom	chromStart	chromEnd
Idua	On target site	**CTTTGAGACCTCTGCCCAGAGTTGNNNNCC**	**CTTTGAGACCTCTGCCCAGAGTTGTTCTCC**	TRUE		***Idua***	**100**	**0**		....	**1**	**chr5:108681382-108681411**	**-**	**chr5**	**108681382**	**108681411**
	1	CTTTGAGACCTCTGCCCAGAGTTGNNNNCC	ATGTGAGTACTGTCCCCAGAGTTGGGGTCC				0.6	6	24,22,17,16,13,11	A.G....TA..G.C..........	1	chr1:126987167-126987196	+	chr1	126987167	126987196
	2	CTTTGAGACCTCTGCCCAGAGTTGNNNNCC	CCTGAAAGGCTCTGCCCAGAGTTGTGGCCC				0.6	6	23,21,20,18,17,16	.C.GA.AGG...............	1	chr9:43593905-43593934	+	chr9	43593905	43593934
	3	CTTTGAGACCTCTGCCCAGAGTTGNNNNCC	CTGAGCTCCCTCTCCCCAGAGTTGTCAGCC		TRUE	*Amph*	0.6	6	22,21,19,18,17,11	..GA.CTC.....C..........	1	chr13:19013688-19013717	-	chr13	19013688	19013717
	4	CTTTGAGACCTCTGCCCAGAGTTGNNNNCC	ATTTGGGAGCTGTTCCCAGAGTTCAAACCC		TRUE	*Acot12*	0.3	6	24,19,16,13,11,1	A....G..G..G.T.........C	1	chr13:91772997-91773026	-	chr13	91772997	91773026
	5	CTTTGAGACCTCTGCCCAGAGTTGNNNNCC	CTAGCAGAACTTTGCCCAGACTTGCTGACC				0.3	6	22,21,20,16,13,4	..AGC...A..T........C...	1	chr3:21762332-21762361	+	chr3	21762332	21762361

Top gene ontology categories (FDR <= 5%) for significantly differentially expressed genes in [dual-sgRNA.Design 1 vs PBS]. All tests were done using an iterative gene ontology analysis (Methods).
GO.NAMESPACE=biological_process

GO.ID	GO.NAME	N.TEST	CAT.N.FG.GENE	CAT.N.BG.GENE	N.FG.GENE	N.BG.GENE	P	ODDS.RATIO
GO:0044281	small molecule metabolic process	6126	377	556	2767	6629	1.23E-11	1.62
GO:0009605	response to external stimulus	5337	214	370	2390	6073	2.48E-05	1.47
GO:0006351	transcription, DNA-templated	4484	195	716	2176	5703	4.81E-05	0.714
GO:0010951	negative regulation of endopeptidase activity	4050	39	38	1981	4987	6.08E-05	2.58
GO:0015721	bile acid and bile salt transport	3992	7	1	1942	4949	0.000859	17.8
GO:0033138	positive regulation of peptidyl-serine phosphorylation	3982	0	22	1935	4948	0.00123	0
GO:0071103	DNA conformation change	3949	3	39	1935	4926	0.00152	0.196
GO:0007010	cytoskeleton organization	3917	65	255	1932	4887	0.00174	0.645
GO:0045445	myoblast differentiation	3656	5	0	1867	4632	0.00197	inf
GO:0030516	regulation of axon extension	3645	20	18	1862	4632	0.00198	2.76
GO:0009913	epidermal cell differentiation	3602	10	5	1842	4614	0.00245	5.01
GO:0031638	zymogen activation	3578	9	4	1832	4609	0.00272	5.66
GO:0019835	cytolysis	3567	6	1	1823	4605	0.00278	15.2
GO:1901136	carbohydrate derivative catabolic process	3563	12	8	1817	4604	0.00431	3.8
GO:0002285	lymphocyte activation involved in immune response	3546	1	23	1805	4596	0.00554	0.111
GO:0030041	actin filament polymerization	3492	5	1	1804	4573	0.00835	12.7
GO:0010984	regulation of lipoprotein particle clearance	3489	5	1	1799	4572	0.00828	12.7
GO:0048545	response to steroid hormone	3480	15	14	1794	4571	0.0112	2.73
GO:0042634	regulation of hair cycle	3424	5	1	1779	4557	0.00806	12.8
GO:1905330	regulation of morphogenesis of an epithelium	3404	0	21	1774	4556	0.00218	0
GO:0030100	regulation of endocytosis	3375	23	27	1774	4535	0.00711	2.18
GO:0051049	regulation of transport	3309	102	353	1751	4508	0.0106	0.744
GO:1903037	regulation of leukocyte cell-cell adhesion	2739	20	20	1649	4155	0.00436	2.52
GO:0034109	homotypic cell-cell adhesion	2677	7	3	1629	4135	0.00748	5.92
GO:1901796	regulation of signal transduction by p53 class mediator	2671	6	2	1622	4132	0.00816	7.64

GO:0048741	skeletal muscle fiber development	2664	5	1	1616	4130	0.00813	12.8
GO:0006820	anion transport	2655	44	67	1611	4129	0.0103	1.68
GO:0042593	glucose homeostasis	2587	8	5	1567	4062	0.0115	4.15
GO:0000479	endonucleolytic cleavage of tricistronic rRNA transcript (SSU-rRNA, 5.8S rRNA, LSU-rRNA)	2568	5	2	1559	4057	0.0206	6.51
GO:0045824	negative regulation of innate immune response	2564	5	2	1554	4055	0.0204	6.52
GO:0006890	retrograde vesicle-mediated transport, Golgi to ER	2552	0	13	1549	4053	0.0256	0
GO:0045185	maintenance of protein location	2549	8	6	1549	4040	0.0302	3.48
GO:0009953	dorsal/ventral pattern formation	2540	9	8	1541	4034	0.0283	2.95
GO:0033365	protein localization to organelle	2521	28	116	1532	4026	0.0296	0.634
GO:1901361	organic cyclic compound catabolic process	2407	25	35	1504	3910	0.0205	1.86
GO:2000785	regulation of autophagosome assembly	2375	5	2	1479	3875	0.0202	6.55
GO:0006888	ER to Golgi vesicle-mediated transport	2371	1	19	1474	3873	0.0222	0.138
GO:0090305	nucleic acid phosphodiester bond hydrolysis	2369	9	52	1473	3854	0.0294	0.453
GO:0055088	lipid homeostasis	2346	5	2	1464	3802	0.0208	6.49
GO:0016042	lipid catabolic process	2338	15	18	1459	3800	0.0311	2.17
GO:0071345	cellular response to cytokine stimulus	2321	10	9	1444	3782	0.0207	2.91
GO:0002920	regulation of humoral immune response	2305	4	1	1434	3773	0.0225	10.5
GO:0044089	positive regulation of cellular component biogenesis	2290	11	60	1430	3772	0.0226	0.484
GO:0051960	regulation of nervous system development	2222	40	62	1419	3712	0.0137	1.69
GO:0043087	regulation of GTPase activity	2099	20	94	1379	3650	0.0189	0.563
GO:0071417	cellular response to organonitrogen compound	2045	0	17	1359	3556	0.00562	0
GO:0019932	second-messenger-mediated signaling	2009	9	6	1359	3539	0.00899	3.91
GO:0071407	cellular response to organic cyclic compound	1989	6	3	1350	3533	0.0169	5.23
GO:0070085	glycosylation	1980	16	81	1344	3530	0.0153	0.519
GO:0007369	gastrulation	1947	6	3	1328	3449	0.0174	5.19

GO:0042246	tissue regeneration	1936	5	2	1322	3446	0.0205	6.52
GO:0090199	regulation of release of cytochrome c from mitochondria	1931	4	1	1317	3444	0.0229	10.5
GO:0048469	cell maturation	1926	9	8	1313	3443	0.0281	2.95
GO:1902806	regulation of cell cycle G1/S phase transition	1915	8	7	1304	3435	0.0387	3.01
GO:0006468	protein phosphorylation	1904	31	126	1296	3428	0.0357	0.651
GO:0046328	regulation of JNK cascade	1817	9	6	1265	3302	0.00889	3.92
GO:0008380	RNA splicing	1802	17	87	1256	3296	0.0102	0.513
GO:0007286	spermatid development	1766	1	19	1239	3209	0.022	0.136
GO:0097435	supramolecular fiber organization	1759	9	8	1238	3190	0.0299	2.9
GO:0034329	cell junction assembly	1738	9	8	1229	3182	0.0294	2.91
GO:0042391	regulation of membrane potential	1727	0	11	1220	3174	0.0417	0
GO:0070228	regulation of lymphocyte apoptotic process	1715	4	1	1220	3163	0.0234	10.4
GO:0098609	cell-cell adhesion	1708	5	34	1216	3162	0.046	0.382
GO:0000209	protein polyubiquitination	1688	11	54	1211	3128	0.0509	0.526
GO:0042787	protein ubiquitination involved in ubiquitin-dependent protein catabolic process	1667	14	15	1200	3074	0.0218	2.39
GO:0048858	cell projection morphogenesis	1659	1	17	1186	3059	0.0342	0.152
GO:0006338	chromatin remodeling	1642	1	15	1185	3042	0.0537	0.171
GO:0018205	peptidyl-lysine modification	1629	6	35	1184	3027	0.0559	0.438
GO:0042176	regulation of protein catabolic process	1597	4	32	1178	2992	0.024	0.317
GO:1903320	regulation of protein modification by small protein conjugation or removal	1565	13	14	1174	2960	0.0313	2.34
GO:0048869	cellular developmental process	1546	89	171	1161	2946	0.0404	1.32
GO:0006354	DNA-templated transcription, elongation	1359	4	1	1072	2775	0.0236	10.4
GO:0001503	ossification	1358	4	1	1068	2774	0.0234	10.4
GO:0001890	placenta development	1352	4	1	1064	2773	0.0231	10.4
GO:0031401	positive regulation of protein modification process	1347	6	39	1060	2772	0.0296	0.402
GO:0051248	negative regulation of protein metabolic process	1307	25	34	1054	2733	0.0187	1.91

GO:0002250	adaptive immune response	1247	8	5	1029	2699	0.0109	4.2
GO:0031214	biomineral tissue development	1240	4	1	1021	2694	0.0224	10.6
GO:0032007	negative regulation of TOR signaling	1239	4	1	1017	2693	0.0222	10.6
GO:0007165	signal transduction	1234	72	264	1013	2692	0.0205	0.725
GO:1903706	regulation of hemopoiesis	1087	0	12	941	2428	0.0255	0
GO:0003012	muscle system process	1077	0	12	941	2416	0.0253	0
GO:0000387	spliceosomal snRNP assembly	1067	0	11	941	2404	0.0412	0
GO:0048584	positive regulation of response to stimulus	1065	19	25	941	2393	0.0418	1.93
GO:0010212	response to ionizing radiation	1018	5	3	922	2368	0.0443	4.28
GO:0051276	chromosome organization	1012	3	24	917	2365	0.0534	0.322
GO:0007160	cell-matrix adhesion	998	4	2	914	2341	0.0566	5.12
GO:0043062	extracellular structure organization	996	0	16	910	2339	0.00919	0
GO:0006935	chemotaxis	988	4	2	910	2323	0.057	5.11
GO:0055114	oxidation-reduction process	986	60	112	906	2321	0.0565	1.37
GO:0018198	peptidyl-cysteine modification	924	4	1	846	2209	0.0231	10.4
GO:1901214	regulation of neuron death	921	5	3	842	2208	0.0417	4.37
GO:0051345	positive regulation of hydrolase activity	913	0	11	837	2205	0.0421	0
GO:0042274	ribosomal small subunit biogenesis	903	4	2	837	2194	0.0535	5.24
GO:1901566	organonitrogen compound biosynthetic process	901	31	123	833	2192	0.0507	0.663
GO:0016072	rRNA metabolic process	867	21	29	802	2069	0.038	1.87
GO:0006793	phosphorus metabolic process	858	35	57	781	2040	0.0336	1.6
GO:0051247	positive regulation of protein metabolic process	817	8	7	746	1983	0.0379	3.04
GO:0000910	cytokinesis	802	0	10	738	1976	0.0711	0
GO:0006302	double-strand break repair	800	6	5	738	1966	0.0813	3.2

Top gene ontology categories (FDR <= 5%) for significantly differentially expressed genes in [dual-sgRNA.Design 4 vs PBS]. All tests were done using an iterative gene ontology analysis (Methods). GO.NAMESPACE=biological_process

GO.ID	GO.NAME	N.TEST	CAT.N.FG.GENE	CAT.N.BG.GENE	N.FG.GENE	N.BG.GENE	P	ODDS.RATIO
GO:0019752	carboxylic acid metabolic process	6169	216	287	2831	6710	1.00E-09	1.78
GO:0052548	regulation of endopeptidase activity	5805	86	111	2615	6423	1.63E-05	1.9
GO:0006721	terpenoid metabolic process	5622	15	7	2529	6312	0.000139	5.35
GO:0035458	cellular response to interferon-beta	5577	11	3	2514	6305	0.000151	9.2
GO:0007035	vacuolar acidification	5564	8	1	2503	6302	0.00029	20.1
GO:1901796	regulation of signal transduction by p53 class mediator	5557	17	10	2495	6301	0.000311	4.29
GO:0046677	response to antibiotic	5503	14	8	2478	6291	0.000593	4.44
GO:0045824	negative regulation of innate immune response	5474	12	6	2464	6283	0.000782	5.1
GO:0002040	sprouting angiogenesis	5445	10	4	2452	6277	0.000958	6.4
GO:0045454	cell redox homeostasis	5413	18	14	2442	6273	0.0011	3.3
GO:0032869	cellular response to insulin stimulus	5400	20	17	2424	6259	0.00133	3.04
GO:0042391	regulation of membrane potential	5352	19	109	2404	6242	0.000922	0.453
GO:0000447	endonucleolytic cleavage in ITS1 to separate SSU-rRNA from 5.8S rRNA and LSU-rRNA from tricistronic rRNA transcript (SSU-rRNA, 5.8S rRNA, LSU-rRNA)	5166	5	0	2385	6133	0.00173	inf
GO:0032886	regulation of microtubule-based process	5162	14	84	2380	6133	0.00204	0.429
GO:0016126	sterol biosynthetic process	5065	10	5	2366	6049	0.00217	5.11
GO:0070613	regulation of protein processing	5054	14	10	2356	6044	0.0022	3.59
GO:0031295	T cell costimulation	5032	9	4	2342	6034	0.00238	5.8
GO:0002312	B cell activation involved in immune response	5016	0	25	2333	6030	0.000455	0
GO:0071157	negative regulation of cell cycle arrest	4975	6	1	2333	6005	0.00257	15.4
GO:0006351	transcription, DNA-templated	4970	223	732	2327	6004	0.00249	0.786
GO:0097193	intrinsic apoptotic signaling pathway	4481	20	16	2104	5272	0.00071	3.13
GO:0006970	response to osmotic stress	4449	11	6	2084	5256	0.00202	4.62
GO:0090278	negative regulation of peptide hormone secretion	4430	0	18	2073	5250	0.0033	0
GO:0061028	establishment of endothelial barrier	4406	0	17	2073	5232	0.00538	0
GO:0051279	regulation of release of sequestered calcium ion into cytosol	4367	10	6	2073	5215	0.00474	4.19
GO:0043901	negative regulation of multi-organism process	4331	24	27	2063	5209	0.00476	2.24
GO:0006468	protein phosphorylation	4238	63	244	2039	5182	0.00277	0.656
GO:0031099	regeneration	3939	20	17	1976	4938	0.00147	2.94

GO:0015721	bile acid and bile salt transport	3900	6	2	1956	4921	0.00855	7.55
GO:0044062	regulation of excretion	3898	6	2	1950	4919	0.00846	7.57
GO:0010984	regulation of lipoprotein particle clearance	3876	5	1	1944	4917	0.00841	12.6
GO:0061077	chaperone-mediated protein folding	3870	1	20	1939	4916	0.0137	0.127
GO:0070192	chromosome organization involved in meiotic cell cycle	3859	1	20	1938	4896	0.0137	0.126
GO:0071346	cellular response to interferon-gamma	3831	7	4	1937	4876	0.0161	4.41
GO:0030041	actin filament polymerization	3818	5	1	1930	4872	0.00847	12.6
GO:0010562	positive regulation of phosphorus metabolic process	3817	50	191	1925	4871	0.0105	0.662
GO:1901136	carbohydrate derivative catabolic process	3495	20	20	1875	4680	0.00456	2.5
GO:0043666	regulation of phosphoprotein phosphatase activity	3444	5	1	1855	4660	0.00861	12.6
GO:0014070	response to organic cyclic compound	3442	51	82	1850	4659	0.0151	1.57
GO:0060070	canonical Wnt signaling pathway	3257	0	18	1799	4577	0.00334	0
GO:0006855	drug transmembrane transport	3205	16	15	1799	4559	0.00797	2.7
GO:0048536	spleen development	3178	5	1	1783	4544	0.0082	12.7
GO:0006111	regulation of gluconeogenesis	3174	7	4	1778	4543	0.0151	4.47
GO:0032269	negative regulation of cellular protein metabolic process	3166	58	98	1771	4539	0.015	1.52
GO:0044092	negative regulation of molecular function	3029	15	85	1713	4441	0.00329	0.458
GO:0048771	tissue remodeling	2939	7	4	1698	4356	0.0149	4.49
GO:0099517	synaptic vesicle transport along microtubule	2903	6	3	1691	4352	0.0179	5.15
GO:0051649	establishment of localization in cell	2893	89	320	1685	4349	0.0071	0.718
GO:0033119	negative regulation of RNA splicing	2660	8	4	1596	4029	0.00663	5.05
GO:0018198	peptidyl-cysteine modification	2655	5	2	1588	4025	0.0224	6.34
GO:0034330	cell junction organization	2650	15	16	1583	4023	0.0167	2.38
GO:0050678	regulation of epithelial cell proliferation	2623	1	21	1568	4007	0.0146	0.122
GO:0030154	cell differentiation	2611	103	188	1567	3986	0.0094	1.39
GO:0034654	nucleobase-containing compound biosynthetic process	2435	49	80	1464	3798	0.0131	1.59
GO:0051259	protein oligomerization	2309	29	37	1415	3718	0.0052	2.06
GO:0006874	cellular calcium ion homeostasis	2262	2	30	1386	3681	0.00473	0.177
GO:0001525	angiogenesis	2238	13	13	1384	3651	0.0148	2.64
GO:0006955	immune response	2213	43	72	1371	3638	0.0202	1.58
GO:0051704	multi-organism process	2114	13	75	1328	3566	0.0103	0.465

GO:0006497	protein lipidation	2031	4	35	1315	3491	0.0174	0.303
GO:0009719	response to endogenous stimulus	2020	4	35	1311	3456	0.0175	0.301
GO:0055002	striated muscle cell development	1984	4	1	1307	3421	0.0228	10.5
GO:0072523	purine-containing compound catabolic process	1977	4	1	1303	3420	0.0227	10.5
GO:0001890	placenta development	1974	4	1	1299	3419	0.0225	10.5
GO:0032007	negative regulation of TOR signaling	1971	4	1	1295	3418	0.0223	10.6
GO:0002181	cytoplasmic translation	1970	9	8	1291	3417	0.0273	2.98
GO:0051438	regulation of ubiquitin-protein transferase activity	1968	6	4	1282	3409	0.0307	3.99
GO:0051963	regulation of synapse assembly	1960	0	12	1276	3405	0.0447	0
GO:0051568	histone H3-K4 methylation	1956	4	2	1276	3393	0.0515	5.32
GO:0008284	positive regulation of cell proliferation	1948	7	42	1272	3391	0.0511	0.444
GO:0007292	female gamete generation	1892	4	1	1265	3349	0.0222	10.6
GO:0006281	DNA repair	1890	16	79	1261	3348	0.0261	0.538
GO:0001775	cell activation	1833	11	12	1245	3269	0.0366	2.41
GO:1903037	regulation of leukocyte cell-cell adhesion	1817	5	3	1234	3257	0.0408	4.4
GO:0048870	cell motility	1797	16	74	1229	3254	0.0422	0.572
GO:1900180	regulation of protein localization to nucleus	1711	0	15	1213	3180	0.0162	0
GO:0010771	negative regulation of cell morphogenesis involved in differentiation	1684	5	2	1213	3165	0.0205	6.52
GO:0048638	regulation of developmental growth	1677	11	11	1208	3163	0.0291	2.62
GO:0031214	biomineral tissue development	1642	5	3	1197	3152	0.041	4.39
GO:0019932	second-messenger-mediated signaling	1640	5	3	1192	3149	0.0406	4.4
GO:0022603	regulation of anatomical structure morphogenesis	1634	8	45	1187	3146	0.0444	0.471
GO:0001503	ossification	1580	5	3	1179	3101	0.0412	4.38
GO:0006639	acylglycerol metabolic process	1575	7	6	1174	3098	0.0554	3.08
GO:0050817	coagulation	1556	5	4	1167	3092	0.0705	3.31
GO:0006730	one-carbon metabolic process	1547	5	4	1162	3088	0.0699	3.32
GO:0031365	N-terminal protein amino acid modification	1545	5	4	1157	3084	0.0693	3.33
GO:0019439	aromatic compound catabolic process	1542	8	42	1152	3080	0.0796	0.509
GO:0050792	regulation of viral process	1500	4	2	1144	3038	0.0517	5.31
GO:0045185	maintenance of protein location	1495	5	4	1140	3036	0.0695	3.33
GO:0007399	nervous system development	1490	6	5	1135	3032	0.0811	3.21
GO:0009790	embryo development	1485	2	19	1129	3027	0.0843	0.282
GO:1903510	mucopolysaccharide metabolic process	1470	1	14	1127	3008	0.0846	0.191

GO:0035269	protein O-linked mannosylation	1457	4	3	1126	2994	0.0945	3.55
GO:0035082	axoneme assembly	1453	4	3	1122	2991	0.0939	3.55
GO:0009056	catabolic process	1449	68	141	1118	2988	0.0957	1.29
GO:1905114	cell surface receptor signaling pathway involved in cell-cell signaling	1349	1	19	1050	2847	0.0229	0.143
GO:0031401	positive regulation of protein modification process	1341	0	12	1049	2828	0.0444	0
GO:0030111	regulation of Wnt signaling pathway	1325	6	4	1049	2816	0.0297	4.03
GO:0007186	G-protein coupled receptor signaling pathway	1318	34	61	1043	2812	0.0625	1.5
GO:0048666	neuron development	1277	6	4	1009	2751	0.0282	4.09

Top gene ontology categories (FDR <= 5%) for significantly differentially expressed genes in [rAAV:HDR:uncleaved vs rAAV:ncDonor.FahSpacer]. All tests were done using an iterative gene ontology analysis (Methods). GO.NAMESPACE biological_process

GO.ID	GO.NAME	N.TEST	CAT.N.FG.GENE	CAT.N.BG.GENE	N.FG.GENE	N.BG.GENE	P	ODDS.RATIO
GO:0044281	small molecule metabolic process	6330	594	380	5080	4832	5.71E-09	1.49
GO:0048872	homeostasis of number of cells	5505	26	65	4486	4452	4.88E-05	0.397
GO:0061041	regulation of wound healing	5363	51	20	4460	4387	0.000306	2.51
GO:0015849	organic acid transport	5238	63	30	4409	4367	0.000791	2.08
GO:0043393	regulation of protein binding	5166	66	34	4346	4337	0.0017	1.94
GO:0045321	leukocyte activation	5014	70	117	4280	4303	0.000841	0.602
GO:1905953	negative regulation of lipid localization	4750	16	2	4210	4186	0.0013	7.95
GO:1904062	regulation of cation transmembrane transport	4718	32	63	4194	4184	0.00182	0.507
GO:0030514	negative regulation of BMP signaling pathway	4583	3	17	4162	4121	0.00142	0.175
GO:0006623	protein targeting to vacuole	4553	0	9	4159	4104	0.00185	0
GO:0051179	localization	4547	945	796	4159	4095	0.0034	1.17
GO:0070988	demethylation	3415	2	17	3214	3299	0.000735	0.121
GO:1901652	response to peptide	3391	18	44	3212	3282	0.00132	0.418
GO:0042327	positive regulation of phosphorylation	3280	149	102	3194	3238	0.00301	1.48
GO:0098869	cellular oxidant detoxification	2994	17	4	3045	3136	0.00388	4.38
GO:0045815	positive regulation of gene expression, epigenetic	2983	0	9	3028	3132	0.00393	0
GO:1900151	regulation of nuclear-transcribed mRNA catabolic process, deadenylation-dependent decay	2976	7	0	3028	3123	0.00704	inf
GO:0003281	ventricular septum development	2967	7	0	3021	3123	0.00698	inf
GO:0030334	regulation of cell migration	2944	97	66	3014	3123	0.00884	1.52
GO:0050673	epithelial cell proliferation	2722	1	13	2917	3057	0.00189	0.0806
GO:0032675	regulation of interleukin-6 production	2707	3	15	2916	3044	0.00773	0.209
GO:0000079	regulation of cyclin-dependent protein serine/threonine kinase activity	2670	9	1	2913	3029	0.0104	9.36
GO:0031648	protein destabilization	2652	9	1	2904	3028	0.0103	9.38
GO:0006120	mitochondrial electron transport, NADH to ubiquinone	2643	6	0	2895	3027	0.0137	inf
GO:0021675	nerve development	2641	0	7	2889	3027	0.0158	0

GO:0007186	G-protein coupled receptor signaling pathway	2633	54	86	2889	3020	0.0165	0.656
GO:0051291	protein heterooligomerization	2538	15	4	2835	2934	0.0106	3.88
GO:0043279	response to alkaloid	2523	12	3	2820	2930	0.0192	4.16
GO:0051896	regulation of protein kinase B signaling	2511	8	1	2808	2927	0.0192	8.34
GO:0035019	somatic stem cell population maintenance	2507	1	9	2800	2926	0.0217	0.116
GO:1902808	positive regulation of cell cycle G1/S phase transition	2496	7	0	2799	2917	0.00679	inf
GO:0051726	regulation of cell cycle	2494	94	134	2792	2917	0.0256	0.733
GO:0030239	myofibril assembly	2279	0	8	2698	2783	0.00784	0
GO:0061077	chaperone-mediated protein folding	2273	13	3	2698	2775	0.0117	4.46
GO:0032784	regulation of DNA-templated transcription, elongation	2268	13	3	2685	2772	0.0115	4.47
GO:0051289	protein homotetramerization	2256	8	1	2672	2769	0.0195	8.29
GO:0003013	circulatory system process	2255	8	1	2664	2768	0.0194	8.31
GO:0051241	negative regulation of multicellular organismal process	2239	56	87	2656	2767	0.022	0.671
GO:0031110	regulation of microtubule polymerization or depolymerization	2081	9	1	2600	2680	0.0107	9.28
GO:0007059	chromosome segregation	2070	8	23	2591	2679	0.0109	0.36
GO:0043062	extracellular structure organization	2056	29	13	2583	2656	0.0126	2.29
GO:0045103	intermediate filament-based process	2032	6	0	2554	2643	0.0141	inf
GO:0034332	adherens junction organization	2030	6	0	2548	2643	0.014	inf
GO:0008213	protein alkylation	2020	35	18	2542	2643	0.0181	2.02
GO:0007049	cell cycle	1991	46	27	2507	2625	0.0181	1.78
GO:0008202	steroid metabolic process	1974	15	5	2461	2598	0.0235	3.17
GO:0042632	cholesterol homeostasis	1970	5	0	2446	2593	0.027	inf
GO:0018198	peptidyl-cysteine modification	1966	0	6	2441	2593	0.0316	0
GO:0007423	sensory organ development	1959	3	12	2441	2587	0.0362	0.265
GO:0043523	regulation of neuron apoptotic process	1938	4	14	2438	2575	0.0318	0.302
GO:0048545	response to steroid hormone	1928	17	6	2434	2561	0.0201	2.98
GO:0043484	regulation of RNA splicing	1904	27	14	2417	2555	0.0288	2.04
GO:0000413	protein peptidyl-prolyl isomerization	1881	2	10	2390	2541	0.0395	0.213
GO:0051248	negative regulation of protein metabolic process	1878	89	66	2388	2531	0.0336	1.43

GO:0070192	chromosome organization involved in meiotic cell cycle	1740	0	8	2299	2465	0.00806	0
GO:0009887	animal organ morphogenesis	1732	14	32	2299	2457	0.0173	0.468
GO:0048468	cell development	1682	49	31	2285	2425	0.0244	1.68
GO:0042176	regulation of protein catabolic process	1619	30	16	2236	2394	0.0257	2.01
GO:0002250	adaptive immune response	1581	4	15	2206	2378	0.0207	0.287
GO:0043462	regulation of ATPase activity	1567	0	6	2202	2363	0.0318	0
GO:0034612	response to tumor necrosis factor	1563	7	1	2202	2357	0.0335	7.49
GO:0002573	myeloid leukocyte differentiation	1554	0	6	2195	2356	0.0318	0
GO:0070085	glycosylation	1552	34	56	2195	2350	0.0548	0.65
GO:0006672	ceramide metabolic process	1524	14	5	2161	2294	0.0368	2.97
GO:0016071	mRNA metabolic process	1514	78	59	2147	2289	0.056	1.41
GO:0045927	positive regulation of growth	1476	11	2	2069	2230	0.0104	5.93
GO:0036211	protein modification process	1453	272	246	2058	2228	0.0564	1.2
GO:0032088	negative regulation of NF-kappaB transcription factor activity	1235	6	0	1786	1982	0.0114	inf
GO:0035304	regulation of protein dephosphorylation	1231	6	0	1780	1982	0.0113	inf
GO:0060341	regulation of cellular localization	1229	11	28	1774	1982	0.0229	0.439
GO:2001020	regulation of response to DNA damage stimulus	1184	7	1	1763	1954	0.0312	7.76
GO:0002768	immune response-regulating cell surface receptor signaling pathway	1175	0	6	1756	1953	0.0325	0
GO:0002683	negative regulation of immune system process	1164	3	12	1756	1947	0.0387	0.277
GO:0003341	cilium movement	1153	3	12	1753	1935	0.0384	0.276
GO:0032392	DNA geometric change	1145	3	12	1750	1923	0.038	0.275
GO:0030178	negative regulation of Wnt signaling pathway	1134	8	1	1747	1911	0.017	8.75
GO:0098742	cell-cell adhesion via plasma-membrane adhesion molecules	1129	12	26	1739	1910	0.0511	0.507
GO:0005975	carbohydrate metabolic process	1114	18	8	1727	1884	0.0314	2.45
GO:0001894	tissue homeostasis	1094	8	2	1709	1876	0.0557	4.39
GO:0006359	regulation of transcription by RNA polymerase III	1085	6	1	1701	1874	0.059	6.61
GO:0050953	sensory perception of light stimulus	1079	3	11	1695	1873	0.0615	0.301
GO:0046856	phosphatidylinositol dephosphorylation	1069	0	5	1692	1862	0.0638	0
GO:0071229	cellular response to acid chemical	1067	0	5	1692	1857	0.0638	0

GO:0043241	protein complex disassembly	1064	2	9	1692	1852	0.0682	0.243
GO:0008285	negative regulation of cell proliferation	1060	6	16	1690	1843	0.0571	0.409
GO:0051603	proteolysis involved in cellular protein catabolic process	1045	14	28	1684	1827	0.0633	0.542
GO:0019882	antigen processing and presentation	1021	6	0	1670	1799	0.0125	inf
GO:0048731	system development	1020	19	10	1664	1799	0.0644	2.05
GO:0120034	positive regulation of plasma membrane bounded cell projection assembly	1009	2	9	1645	1789	0.0678	0.242
GO:0006360	transcription by RNA polymerase I	994	1	7	1643	1780	0.0719	0.155
GO:0009451	RNA modification	993	15	28	1642	1773	0.0924	0.578
GO:0010033	response to organic substance	981	41	29	1627	1745	0.0918	1.52
GO:0045892	negative regulation of transcription, DNA-templated	920	51	75	1586	1716	0.102	0.736
GO:0009890	negative regulation of biosynthetic process	872	8	2	1535	1641	0.0579	4.28
GO:0007163	establishment or maintenance of cell polarity	861	6	1	1527	1639	0.0618	6.44
GO:0045165	cell fate commitment	858	0	5	1521	1638	0.0632	0
GO:0043086	negative regulation of catalytic activity	857	10	22	1521	1633	0.074	0.488
GO:0006284	base-excision repair	838	0	5	1511	1611	0.063	0
GO:1903706	regulation of hemopoiesis	837	2	8	1511	1606	0.111	0.266
GO:0019221	cytokine-mediated signaling pathway	821	9	3	1509	1598	0.0844	3.18

Top gene ontology categories (FDR <= 5%) for significantly differentially expressed genes in [rAAV:HDR:cleaved vs rAAV:ncDonor.FahSpacer]. All tests were done using an iterative gene ontology analysis (Methods). GO.NAMESPACE biological_process

GO.ID	GO.NAME	N.TEST	CAT.N.FG.GENE	CAT.N.BG.GENE	N.FG.GENE	N.BG.GENE	P	ODDS.RATIO
GO:0044281	small molecule metabolic process	6360	557	419	4738	5340	2.14E-09	1.5
GO:0045721	negative regulation of gluconeogenesis	5545	11	0	4181	4921	0.000194	inf
GO:0050820	positive regulation of coagulation	5538	16	2	4170	4921	0.000208	9.44
GO:0072698	protein localization to microtubule cytoskeleton	5501	18	3	4154	4919	0.000239	7.1
GO:0048872	homeostasis of number of cells	5478	24	65	4136	4916	0.000373	0.439
GO:0051924	regulation of calcium ion transport	5341	29	71	4112	4851	0.000788	0.482
GO:0050953	sensory perception of light stimulus	5174	10	37	4083	4780	0.000605	0.316
GO:0097190	apoptotic signaling pathway	5144	87	60	4073	4743	0.00196	1.69
GO:0006623	protein targeting to vacuole	4997	0	10	3986	4683	0.00254	0
GO:0043280	positive regulation of cysteine-type endopeptidase activity involved in apoptotic process	4988	8	29	3986	4673	0.00269	0.323
GO:0010038	response to metal ion	4934	69	44	3978	4644	0.00167	1.83
GO:0045815	positive regulation of gene expression, epigenetic	4798	1	12	3909	4600	0.00473	0.0981
GO:0006337	nucleosome disassembly	4792	7	0	3908	4588	0.00437	inf
GO:0055114	oxidation-reduction process	4782	145	119	3901	4588	0.00471	1.43
GO:0052548	regulation of endopeptidase activity	4700	64	43	3756	4469	0.00442	1.77
GO:0030574	collagen catabolic process	4623	0	11	3692	4426	0.00144	0
GO:0022407	regulation of cell-cell adhesion	4610	41	83	3692	4415	0.00621	0.591
GO:0034109	homotypic cell-cell adhesion	4372	21	8	3651	4332	0.00462	3.11
GO:0016192	vesicle-mediated transport	4347	242	221	3630	4324	0.00621	1.3
GO:0031175	neuron projection development	4115	18	50	3388	4103	0.00202	0.436
GO:0010975	regulation of neuron projection development	4038	90	64	3370	4053	0.0014	1.69
GO:0007409	axonogenesis	3892	6	25	3280	3989	0.00355	0.292
GO:0006461	protein complex assembly	3862	131	110	3274	3964	0.00569	1.44
GO:0043604	amide biosynthetic process	3656	105	80	3143	3854	0.00162	1.61
GO:0050685	positive regulation of mRNA processing	3621	12	2	3038	3774	0.00223	7.45
GO:0007565	female pregnancy	3609	9	1	3026	3772	0.00686	11.2
GO:0016578	histone deubiquitination	3593	9	1	3017	3771	0.00684	11.2
GO:0030855	epithelial cell differentiation	3590	28	64	3008	3770	0.008	0.548
GO:0017157	regulation of exocytosis	3465	16	5	2980	3706	0.00398	3.98

GO:0009798	axis specification	3432	17	6	2964	3701	0.00556	3.54
GO:0007178	transmembrane receptor protein serine/threonine kinase signaling pathway	3403	10	33	2947	3695	0.00527	0.38
GO:0010712	regulation of collagen metabolic process	3351	6	0	2937	3662	0.0078	inf
GO:0007155	cell adhesion	3346	62	119	2931	3662	0.00628	0.651
GO:0023052	signaling	3239	18	48	2869	3543	0.00414	0.463
GO:0007173	epidermal growth factor receptor signaling pathway	3163	9	1	2851	3495	0.00699	11
GO:0045834	positive regulation of lipid metabolic process	3157	16	6	2842	3494	0.00977	3.28
GO:0030104	water homeostasis	3111	0	8	2826	3488	0.0103	0
GO:0042832	defense response to protozoan	3104	0	8	2826	3480	0.0102	0
GO:0009566	fertilization	3085	3	16	2826	3472	0.0106	0.23
GO:0014070	response to organic cyclic compound	3071	67	51	2823	3456	0.0116	1.61
GO:0001822	kidney development	2919	4	22	2756	3405	0.00246	0.225
GO:0014743	regulation of muscle hypertrophy	2892	6	0	2752	3383	0.00818	inf
GO:1903322	positive regulation of protein modification by small protein conjugation or removal	2870	11	34	2746	3383	0.00636	0.399
GO:0032268	regulation of cellular protein metabolic process	2825	268	262	2735	3349	0.014	1.25
GO:0051276	chromosome organization	2387	34	75	2467	3087	0.00622	0.567
GO:0060249	anatomical structure homeostasis	2299	12	2	2433	3012	0.00226	7.43
GO:0071356	cellular response to tumor necrosis factor	2291	6	0	2421	3010	0.00788	inf
GO:0015850	organic hydroxy compound transport	2284	17	7	2415	3010	0.0124	3.03
GO:0016071	mRNA metabolic process	2269	103	89	2398	3003	0.012	1.45
GO:0051170	nuclear import	2181	20	10	2295	2914	0.0158	2.54
GO:0070873	regulation of glycogen metabolic process	2158	5	0	2275	2904	0.0164	inf
GO:0048869	cellular developmental process	2153	231	234	2270	2904	0.0169	1.26
GO:0031099	regeneration	1836	8	0	2039	2670	0.00125	inf
GO:0002832	negative regulation of response to biotic stimulus	1829	6	0	2031	2670	0.00654	inf
GO:0030178	negative regulation of Wnt signaling pathway	1826	15	6	2025	2670	0.0133	3.3
GO:0051494	negative regulation of cytoskeleton organization	1805	5	21	2010	2664	0.0161	0.316
GO:0006366	transcription by RNA polymerase II	1766	42	31	2005	2643	0.0168	1.79
GO:0048534	hematopoietic or lymphoid organ development	1739	2	14	1963	2612	0.0197	0.19
GO:1902533	positive regulation of intracellular signal transduction	1731	33	23	1961	2598	0.0205	1.9

GO:1901361	organic cyclic compound catabolic process	1697	7	1	1928	2575	0.0245	9.35
GO:0031647	regulation of protein stability	1687	20	12	1921	2574	0.0306	2.23
GO:0097502	mannosylation	1662	3	15	1901	2562	0.0304	0.27
GO:0006355	regulation of transcription, DNA-templated	1656	209	341	1898	2547	0.0354	0.822
GO:0002526	acute inflammatory response	1446	6	0	1689	2206	0.0067	inf
GO:0048524	positive regulation of viral process	1441	5	0	1683	2206	0.0153	inf
GO:0051704	multi-organism process	1435	23	57	1678	2206	0.0116	0.53
GO:0006958	complement activation, classical pathway	1342	8	2	1655	2149	0.0252	5.19
GO:0006813	potassium ion transport	1337	4	17	1647	2147	0.0268	0.307
GO:0006413	translational initiation	1332	1	10	1643	2130	0.029	0.13
GO:0048646	anatomical structure formation involved in morphogenesis	1329	10	28	1642	2120	0.033	0.461
GO:0046856	phosphatidylinositol dephosphorylation	1289	0	6	1632	2092	0.0385	0
GO:0044843	cell cycle G1/S phase transition	1282	0	6	1632	2086	0.0384	0
GO:0030866	cortical actin cytoskeleton organization	1278	0	6	1632	2080	0.0382	0
GO:0035023	regulation of Rho protein signal transduction	1272	3	13	1632	2074	0.0455	0.293
GO:0015695	organic cation transport	1261	6	1	1629	2061	0.0491	7.59
GO:0006400	tRNA modification	1255	6	18	1623	2060	0.0656	0.423
GO:0009612	response to mechanical stimulus	1251	0	5	1617	2042	0.071	0
GO:0046323	glucose import	1245	0	5	1617	2037	0.0708	0
GO:0061572	actin filament bundle organization	1240	0	5	1617	2032	0.0706	0
GO:0060284	regulation of cell development	1234	11	4	1617	2027	0.0346	3.45
GO:2000145	regulation of cell motility	1208	18	11	1606	2023	0.0614	2.06
GO:0046887	positive regulation of hormone secretion	1173	0	6	1588	2012	0.0379	0
GO:0007169	transmembrane receptor protein tyrosine kinase signaling pathway	1160	4	15	1588	2006	0.0613	0.337
GO:0070098	chemokine-mediated signaling pathway	1155	0	5	1584	1991	0.0707	0
GO:0071897	DNA biosynthetic process	1151	11	5	1584	1986	0.0747	2.76
GO:0042592	homeostatic process	1144	48	40	1573	1981	0.0645	1.51
GO:0016070	RNA metabolic process	1058	67	62	1525	1941	0.0861	1.38
GO:0019637	organophosphate metabolic process	1022	37	31	1458	1879	0.0845	1.54
GO:0050000	chromosome localization	983	6	2	1421	1848	0.0855	3.9
GO:0008202	steroid metabolic process	975	5	1	1415	1846	0.0917	6.52
GO:1901576	organic substance biosynthetic process	974	18	42	1410	1845	0.0477	0.561
GO:0051205	protein insertion into membrane	941	5	1	1392	1803	0.0922	6.48

GO:0006605	protein targeting	936	8	21	1387	1802	0.0928	0.495
GO:0033993	response to lipid	924	12	7	1379	1781	0.105	2.21
GO:0097035	regulation of membrane lipid distribution	901	0	6	1367	1774	0.0393	0
GO:0050900	leukocyte migration	897	0	5	1367	1768	0.0728	0
GO:0016569	covalent chromatin modification	893	3	11	1367	1763	0.11	0.352
GO:0009411	response to UV	885	5	1	1364	1752	0.0928	6.42
GO:0009792	embryo development ending in birth or egg hatching	882	7	3	1359	1751	0.116	3.01
GO:0071822	protein complex subunit organization	872	2	9	1352	1748	0.128	0.287

Top gene ontology categories (FDR <= 5%) for significantly differentially expressed genes in [rAAV:HDR:uncleaved vs PBS]. All tests were done using an iterative gene ontology analysis (Methods). GO.NAMESPACE biological_process

GO.ID	GO.NAME	N.TEST	CAT.N.FG.GENE	CAT.N.BG.GENE	N.FG.GENE	N.BG.GENE	P	ODDS.RATIO
GO:1901606	alpha-amino acid catabolic process	6347	45	5	4180	5765	3.02E-12	12.4
GO:1901700	response to oxygen-containing compound	6296	395	368	4135	5760	9.68E-08	1.5
GO:0050818	regulation of coagulation	5232	28	8	3740	5392	9.68E-06	5.05
GO:0044281	small molecule metabolic process	5152	368	382	3712	5384	1.27E-05	1.4
GO:0008154	actin polymerization or depolymerization	4603	19	3	3344	5002	1.07E-05	9.47
GO:0050953	sensory perception of light stimulus	4595	7	41	3325	4999	0.000282	0.257
GO:0043604	amide biosynthetic process	4557	51	137	3318	4958	0.000282	0.556
GO:0016049	cell growth	4522	33	18	3267	4821	0.000525	2.71
GO:0001824	blastocyst development	4460	0	14	3234	4803	0.00138	0
GO:0030490	maturation of SSU-rRNA	4458	14	3	3234	4789	0.000653	6.91
GO:0035556	intracellular signal transduction	4456	254	284	3220	4786	0.0015	1.33
GO:0015711	organic anion transport	4065	63	49	2966	4502	0.000605	1.95
GO:0030182	neuron differentiation	3976	18	58	2903	4453	0.00451	0.476
GO:1901988	negative regulation of cell cycle phase transition	3865	29	18	2885	4395	0.00258	2.45
GO:0045815	positive regulation of gene expression, epigenetic	3815	0	11	2856	4377	0.00464	0
GO:0006958	complement activation, classical pathway	3809	13	5	2856	4366	0.00662	3.97
GO:0001525	angiogenesis	3794	50	43	2843	4361	0.00731	1.78
GO:0061515	myeloid cell development	3684	11	3	2793	4318	0.00423	5.67
GO:0048706	embryonic skeletal system development	3668	1	16	2782	4315	0.00453	0.0969
GO:0043086	negative regulation of catalytic activity	3649	112	120	2781	4299	0.00633	1.44
GO:0031629	synaptic vesicle fusion to presynaptic active zone membrane	3468	7	1	2669	4179	0.00724	11
GO:0050796	regulation of insulin secretion	3461	5	27	2662	4178	0.006	0.291
GO:0030810	positive regulation of nucleotide biosynthetic process	3415	6	0	2657	4151	0.00355	inf
GO:0009311	oligosaccharide metabolic process	3402	0	10	2651	4151	0.00852	0
GO:0010715	regulation of extracellular matrix disassembly	3389	5	0	2651	4141	0.00909	inf
GO:0043933	macromolecular complex subunit organization	3381	148	304	2646	4141	0.00817	0.762
GO:0003007	heart morphogenesis	3105	0	12	2498	3837	0.00487	0
GO:0008625	extrinsic apoptotic signaling pathway via death domain receptors	3077	7	1	2498	3825	0.00791	10.7
GO:0045862	positive regulation of proteolysis	3075	18	57	2491	3824	0.00606	0.485

GO:0051149	positive regulation of muscle cell differentiation	2996	15	6	2473	3767	0.00577	3.81
GO:0006367	transcription initiation from RNA polymerase II promoter	2954	12	4	2458	3761	0.00797	4.59
GO:0048041	focal adhesion assembly	2952	5	0	2446	3757	0.00957	inf
GO:0051255	spindle midzone assembly	2944	5	0	2441	3757	0.00951	inf
GO:2001185	regulation of CD8-positive, alpha-beta T cell activation	2939	5	0	2436	3757	0.00945	inf
GO:0050867	positive regulation of cell activation	2913	11	41	2431	3757	0.00666	0.415
GO:0060761	negative regulation of response to cytokine stimulus	2799	5	0	2420	3716	0.00958	inf
GO:0050673	epithelial cell proliferation	2793	11	4	2415	3716	0.014	4.23
GO:0051321	meiotic cell cycle	2776	9	3	2404	3712	0.016	4.63
GO:0045055	regulated exocytosis	2773	17	10	2395	3709	0.0165	2.63
GO:0002683	negative regulation of immune system process	2753	21	61	2378	3699	0.0121	0.536
GO:0032101	regulation of response to external stimulus	2636	53	49	2357	3638	0.0106	1.67
GO:0070555	response to interleukin-1	2538	0	9	2304	3589	0.0147	0
GO:1901184	regulation of ERBB signaling pathway	2532	8	2	2304	3580	0.0175	6.22
GO:0009266	response to temperature stimulus	2527	13	7	2296	3578	0.0217	2.89
GO:0043900	regulation of multi-organism process	2518	24	19	2283	3571	0.028	1.98
GO:0031326	regulation of cellular biosynthetic process	2473	364	672	2259	3552	0.0241	0.852
GO:0045669	positive regulation of osteoblast differentiation	1942	7	0	1895	2880	0.00156	inf
GO:0010628	positive regulation of gene expression	1932	14	5	1888	2880	0.00368	4.27
GO:0042440	pigment metabolic process	1921	6	1	1874	2875	0.0176	9.2
GO:0071103	DNA conformation change	1912	16	9	1868	2874	0.0141	2.74
GO:0002697	regulation of immune effector process	1898	0	9	1852	2865	0.0146	0
GO:0006998	nuclear envelope organization	1873	1	12	1852	2856	0.0209	0.129
GO:0051235	maintenance of location	1869	20	11	1851	2844	0.00543	2.79
GO:0048145	regulation of fibroblast proliferation	1837	0	8	1831	2833	0.026	0
GO:0071345	cellular response to cytokine stimulus	1829	8	3	1831	2825	0.0305	4.11
GO:0006301	postreplication repair	1819	7	2	1823	2822	0.0336	5.42
GO:0051865	protein autoubiquitination	1814	0	14	1816	2820	0.00142	0
GO:0022604	regulation of cell morphogenesis	1812	33	29	1816	2806	0.0355	1.76
GO:0044070	regulation of anion transport	1762	10	5	1783	2777	0.035	3.11
GO:0046341	CDP-diacylglycerol metabolic process	1753	5	1	1773	2772	0.0367	7.82
GO:0032774	RNA biosynthetic process	1749	8	28	1768	2771	0.0405	0.448
GO:0098542	defense response to other organism	1739	19	13	1760	2743	0.0277	2.28
GO:0009607	response to biotic stimulus	1707	1	17	1741	2730	0.00265	0.0922

GO:0006304	DNA modification	1693	0	8	1740	2713	0.0263	0
GO:0051169	nuclear transport	1689	10	5	1740	2705	0.0352	3.11
GO:0007339	binding of sperm to zona pellucida	1683	0	7	1730	2700	0.0477	0
GO:0044804	autophagy of nucleus	1679	7	3	1730	2693	0.0556	3.63
GO:0005975	carbohydrate metabolic process	1673	20	16	1723	2690	0.0579	1.95
GO:0048705	skeletal system morphogenesis	1651	8	3	1703	2674	0.029	4.19
GO:0030097	hemopoiesis	1639	0	8	1695	2671	0.0266	0
GO:0007155	cell adhesion	1634	69	79	1695	2663	0.06	1.37
GO:0072659	protein localization to plasma membrane	1570	12	7	1626	2584	0.0341	2.72
GO:0000281	mitotic cytokinesis	1549	5	1	1614	2577	0.0347	7.98
GO:0045995	regulation of embryonic development	1545	5	1	1609	2576	0.0344	8
GO:0051240	positive regulation of multicellular organismal process	1532	15	45	1604	2575	0.0438	0.535
GO:0008284	positive regulation of cell proliferation	1472	19	10	1589	2530	0.00383	3.03
GO:0033674	positive regulation of kinase activity	1451	14	10	1570	2520	0.0573	2.25
GO:0006914	autophagy	1424	6	23	1556	2510	0.056	0.421
GO:0140029	exocytic process	1411	0	7	1550	2487	0.0487	0
GO:0035148	tube formation	1407	6	1	1550	2480	0.0153	9.6
GO:0060284	regulation of cell development	1402	10	5	1544	2479	0.0323	3.21
GO:0007186	G-protein coupled receptor signaling pathway	1379	27	71	1534	2474	0.0347	0.613
GO:0007204	positive regulation of cytosolic calcium ion concentration	1346	5	0	1507	2403	0.00856	inf
GO:0008277	regulation of G-protein coupled receptor protein signaling pathway	1334	0	8	1502	2403	0.0272	0
GO:0018345	protein palmitoylation	1327	2	13	1502	2395	0.0603	0.245
GO:2001236	regulation of extrinsic apoptotic signaling pathway	1324	6	2	1500	2382	0.062	4.76
GO:0043062	extracellular structure organization	1321	10	6	1494	2380	0.0693	2.66
GO:2000026	regulation of multicellular organismal development	1307	5	21	1484	2374	0.0449	0.381
GO:0030031	cell projection assembly	1285	4	17	1479	2353	0.0741	0.374
GO:2000045	regulation of G1/S transition of mitotic cell cycle	1267	4	1	1475	2336	0.0777	6.33
GO:0060326	cell chemotaxis	1260	4	1	1471	2335	0.0773	6.35
GO:0030855	epithelial cell differentiation	1257	11	7	1467	2334	0.0558	2.5
GO:0000301	retrograde transport, vesicle recycling within Golgi	1248	4	1	1456	2327	0.0762	6.39
GO:0042254	ribosome biogenesis	1247	4	1	1452	2326	0.0758	6.41
GO:0006577	amino-acid betaine metabolic process	1246	4	1	1448	2325	0.0754	6.42

GO:0010605	negative regulation of macromolecule metabolic process	1242	29	29	1444	2324	0.0774	1.61
GO:0050684	regulation of mRNA processing	1180	0	9	1415	2295	0.016	0
GO:0016073	snRNA metabolic process	1174	7	2	1415	2286	0.0321	5.65
GO:0016477	cell migration	1172	5	21	1408	2284	0.0659	0.386
GO:1903532	positive regulation of secretion by cell	1159	6	2	1403	2263	0.0609	4.84

Top gene ontology categories (FDR <= 5%) for significantly differentially expressed genes in [rAAV:HDR:cleaved vs PBS]. All tests were done using an iterative gene ontology analysis (Methods). GO.NAMESPACE biological_process

GO.ID	GO.NAME	N.TEST	CAT.N.FG.GENE	CAT.N.BG.GENE	N.FG.GENE	N.BG.GENE	P	ODDS.RATIO
GO:0019752	carboxylic acid metabolic process	6368	285	239	3810	6310	5.35E-14	1.97
GO:0002253	activation of immune response	5979	78	52	3525	6071	1.13E-07	2.58
GO:0040012	regulation of locomotion	5822	219	236	3447	6019	7.70E-07	1.62
GO:0030728	ovulation	5195	9	0	3228	5783	9.80E-05	inf
GO:0042542	response to hydrogen peroxide	5176	35	23	3219	5783	0.000165	2.73
GO:0034311	diol metabolic process	5095	9	1	3184	5760	0.000629	16.3
GO:2001235	positive regulation of apoptotic signaling pathway	5088	43	37	3175	5759	0.000961	2.11
GO:0009791	post-embryonic development	5011	4	38	3132	5722	0.000253	0.192
GO:0019217	regulation of fatty acid metabolic process	4969	18	8	3128	5684	0.000654	4.09
GO:0045912	negative regulation of carbohydrate metabolic process	4924	1	23	3110	5676	0.000819	0.0794
GO:0022618	ribonucleoprotein complex assembly	4902	28	101	3109	5653	0.00108	0.504
GO:0060670	branching involved in labyrinthine layer morphogenesis	4845	6	0	3081	5552	0.00208	inf
GO:0070828	heterochromatin organization	4836	6	0	3075	5552	0.00206	inf
GO:0051255	spindle midzone assembly	4832	6	0	3069	5552	0.00204	inf
GO:2000243	positive regulation of reproductive process	4830	13	5	3063	5552	0.00208	4.71
GO:0006270	DNA replication initiation	4780	12	4	3050	5547	0.00239	5.46
GO:0006820	anion transport	4777	86	102	3038	5543	0.00428	1.54
GO:0046883	regulation of hormone secretion	4652	16	70	2952	5441	0.000937	0.421
GO:0034104	negative regulation of tissue remodeling	4511	7	0	2936	5371	0.000693	inf
GO:1901699	cellular response to nitrogen compound	4497	54	53	2929	5371	0.00152	1.87
GO:0016024	CDP-diacylglycerol biosynthetic process	4359	5	0	2875	5318	0.00534	inf
GO:0009968	negative regulation of signal transduction	4355	170	236	2870	5318	0.00565	1.33
GO:0008214	protein dealkylation	3955	0	15	2700	5082	0.00211	0
GO:0050776	regulation of immune response	3936	20	78	2700	5067	0.00262	0.481
GO:0061515	myeloid cell development	3757	14	7	2680	4989	0.00451	3.72
GO:0098742	cell-cell adhesion via plasma-membrane adhesion molecules	3732	33	30	2666	4982	0.00503	2.06
GO:0033120	positive regulation of RNA splicing	3702	10	4	2633	4952	0.0082	4.7
GO:0051016	barbed-end actin filament capping	3695	6	1	2623	4948	0.00855	11.3

GO:0055114	oxidation-reduction process	3691	126	174	2617	4947	0.00942	1.37
GO:0006694	steroid biosynthetic process	3590	7	1	2491	4773	0.00314	13.4
GO:0033014	tetrapyrrole biosynthetic process	3585	8	2	2484	4772	0.00415	7.68
GO:0051262	protein tetramerization	3577	4	28	2476	4770	0.00834	0.275
GO:0050819	negative regulation of coagulation	3552	8	3	2472	4742	0.0107	5.12
GO:0010824	regulation of centrosome duplication	3530	2	20	2464	4739	0.012	0.192
GO:1902807	negative regulation of cell cycle G1/S phase transition	3513	10	5	2462	4719	0.0125	3.83
GO:0030490	maturation of SSU-rRNA	3502	14	9	2452	4714	0.0133	2.99
GO:0007193	adenylate cyclase-inhibiting G-protein coupled receptor signaling pathway	3497	9	4	2438	4705	0.0149	4.34
GO:0003008	system process	3488	78	211	2429	4701	0.0132	0.715
GO:1904589	regulation of protein import	3220	1	20	2351	4490	0.00231	0.0955
GO:0043171	peptide catabolic process	3166	7	2	2350	4470	0.0102	6.66
GO:0001822	kidney development	3159	2	20	2343	4468	0.0119	0.191
GO:0006511	ubiquitin-dependent protein catabolic process	3129	39	117	2341	4448	0.0133	0.633
GO:0072659	protein localization to plasma membrane	3073	12	7	2302	4331	0.014	3.23
GO:0031347	regulation of defense response	3061	37	40	2290	4324	0.016	1.75
GO:0006518	peptide metabolic process	2997	41	123	2253	4284	0.0122	0.634
GO:0007143	female meiotic nuclear division	2960	5	1	2212	4161	0.0216	9.41
GO:0022414	reproductive process	2957	96	242	2207	4160	0.0187	0.748
GO:0022402	cell cycle process	2707	88	106	2111	3918	0.00366	1.54
GO:2000573	positive regulation of DNA biosynthetic process	2545	0	15	2023	3812	0.00212	0
GO:0010639	negative regulation of organelle organization	2528	9	43	2023	3797	0.00794	0.393
GO:0006378	mRNA polyadenylation	2469	0	11	2014	3754	0.0109	0
GO:0010390	histone monoubiquitination	2459	6	1	2014	3743	0.00904	11.2
GO:1901654	response to ketone	2452	0	10	2008	3742	0.0184	0
GO:1901659	glycosyl compound biosynthetic process	2430	8	3	2008	3732	0.0209	4.96
GO:0019674	NAD metabolic process	2408	5	1	2000	3729	0.0221	9.32
GO:0090174	organelle membrane fusion	2400	20	17	1995	3728	0.023	2.2
GO:0097150	neuronal stem cell population maintenance	2387	6	2	1975	3711	0.0245	5.64
GO:0044093	positive regulation of molecular function	2377	132	191	1969	3709	0.0264	1.3
GO:0042692	muscle cell differentiation	2141	0	12	1837	3518	0.0112	0
GO:0001568	blood vessel development	2126	11	6	1837	3506	0.018	3.5
GO:0048598	embryonic morphogenesis	2112	8	39	1826	3500	0.0128	0.393
GO:0051250	negative regulation of lymphocyte activation	2060	0	10	1818	3461	0.019	0

GO:0048013	ephrin receptor signaling pathway	2045	0	11	1818	3451	0.0205	0
GO:0032649	regulation of interferon-gamma production	2034	5	1	1818	3440	0.0212	9.46
GO:0001906	cell killing	2020	0	10	1813	3439	0.0188	0
GO:0050673	epithelial cell proliferation	2005	6	2	1813	3429	0.0239	5.67
GO:0042098	T cell proliferation	2000	6	2	1807	3427	0.0237	5.69
GO:0043065	positive regulation of apoptotic process	1993	18	16	1801	3425	0.0295	2.14
GO:0050684	regulation of mRNA processing	1975	2	19	1783	3409	0.0189	0.201
GO:0006613	cotranslational protein targeting to membrane	1967	0	9	1781	3390	0.0324	0
GO:2000116	regulation of cysteine-type endopeptidase activity	1961	5	1	1781	3381	0.021	9.49
GO:0048534	hematopoietic or lymphoid organ development	1951	3	20	1776	3380	0.0447	0.285
GO:0001654	eye development	1927	6	2	1773	3360	0.0238	5.69
GO:0006260	DNA replication	1921	20	20	1767	3358	0.0455	1.9
GO:0071826	ribonucleoprotein complex subunit organization	1913	4	1	1747	3338	0.0507	7.64
GO:0071786	endoplasmic reticulum tubular network organization	1909	4	1	1743	3337	0.0504	7.66
GO:0031648	protein destabilization	1906	4	1	1739	3336	0.0502	7.67
GO:0051149	positive regulation of muscle cell differentiation	1902	7	4	1735	3335	0.0546	3.36
GO:0040029	regulation of gene expression, epigenetic	1892	7	30	1728	3331	0.0556	0.45
GO:0006457	protein folding	1865	12	43	1721	3301	0.0623	0.535
GO:0006605	protein targeting	1849	19	19	1709	3258	0.0581	1.91
GO:0034622	cellular macromolecular complex assembly	1823	23	74	1690	3239	0.0304	0.596
GO:0051053	negative regulation of DNA metabolic process	1769	4	1	1667	3165	0.0514	7.59
GO:0042254	ribosome biogenesis	1768	5	2	1663	3164	0.0523	4.76
GO:0018023	peptidyl-lysine trimethylation	1767	4	1	1658	3162	0.0509	7.63
GO:0001501	skeletal system development	1764	2	15	1654	3161	0.07	0.255
GO:0045667	regulation of osteoblast differentiation	1745	5	2	1652	3146	0.0521	4.76
GO:0031503	protein complex localization	1743	12	10	1647	3144	0.0692	2.29
GO:0034613	cellular protein localization	1731	21	75	1635	3134	0.0119	0.537
GO:0006897	endocytosis	1652	38	45	1614	3059	0.0364	1.6
GO:1903532	positive regulation of secretion by cell	1616	10	6	1576	3014	0.031	3.19
GO:0071695	anatomical structure maturation	1589	0	9	1566	3008	0.0327	0
GO:0060271	cilium assembly	1582	1	12	1566	2999	0.0439	0.16
GO:0030031	cell projection assembly	1578	7	3	1565	2987	0.0382	4.45
GO:0042147	retrograde transport, endosome to Golgi	1564	1	12	1558	2984	0.0439	0.16
GO:0006826	iron ion transport	1560	4	1	1557	2972	0.0508	7.64

GO:0046916	cellular transition metal ion homeostasis	1558	1	12	1553	2971	0.0438	0.159
GO:0090092	regulation of transmembrane receptor protein serine/threonine kinase signaling pathway	1549	4	1	1552	2959	0.0509	7.63
GO:0030595	leukocyte chemotaxis	1547	4	1	1548	2958	0.0507	7.64
GO:0002335	mature B cell differentiation	1539	4	1	1544	2957	0.0504	7.66

Top gene ontology categories (FDR <= 5%) for significantly differentially expressed genes in [rAAV:HDR:uncleaved vs rAAV:FahDonor:ncSpacer]. All tests were done using an iterative gene ontology analysis (Methods). GO.NAMESPACE biological_process

GO.ID	GO.NAME	N.TEST	CAT.N.FG.GENE	CAT.N.BG.GENE	N.FG.GENE	N.BG.GENE	P	ODDS.RATIO
GO:0044281	small molecule metabolic process	6331	590	383	4950	4957	1.93E-10	1.54
GO:0061041	regulation of wound healing	5511	57	18	4360	4574	2.14E-06	3.32
GO:0015849	organic acid transport	5375	67	29	4303	4556	3.33E-05	2.45
GO:0098869	cellular oxidant detoxification	5307	21	4	4236	4527	0.000396	5.61
GO:0007600	sensory perception	5294	50	99	4215	4523	0.000358	0.542
GO:0007030	Golgi organization	5179	11	36	4165	4424	0.000616	0.325
GO:1901379	regulation of potassium ion transmembrane transport	5168	3	19	4154	4388	0.000933	0.167
GO:0051276	chromosome organization	5139	59	106	4151	4369	0.000935	0.586
GO:0010799	regulation of peptidyl-threonine phosphorylation	5030	15	2	4092	4263	0.00108	7.81
GO:0071539	protein localization to centrosome	4980	9	0	4077	4261	0.00161	inf
GO:0010951	negative regulation of endopeptidase activity	4975	62	33	4068	4261	0.00181	1.97
GO:0007219	Notch signaling pathway	4922	16	44	4006	4228	0.00066	0.384
GO:0045216	cell-cell junction organization	4822	43	18	3990	4184	0.000747	2.51
GO:0046890	regulation of lipid biosynthetic process	4759	34	13	3947	4166	0.00117	2.76
GO:0018027	peptidyl-lysine dimethylation	4695	8	0	3913	4153	0.00308	inf
GO:0006355	regulation of transcription, DNA-templated	4686	637	804	3905	4153	0.00291	0.843
GO:0010605	negative regulation of macromolecule metabolic process	3654	190	130	3268	3349	0.000575	1.5
GO:0051016	barbed-end actin filament capping	3334	8	0	3078	3219	0.00328	inf
GO:0048872	homeostasis of number of cells	3330	10	31	3070	3219	0.00245	0.338
GO:0007049	cell cycle	3247	55	29	3060	3188	0.00288	1.98
GO:0043484	regulation of RNA splicing	3219	19	5	3005	3159	0.00342	3.99
GO:0043624	cellular protein complex disassembly	3207	3	17	2986	3154	0.00275	0.186
GO:0006623	protein targeting to vacuole	3195	0	9	2983	3137	0.00398	0
GO:0031647	regulation of protein stability	3187	39	19	2983	3128	0.00544	2.15
GO:0060249	anatomical structure homeostasis	3128	21	7	2944	3109	0.00697	3.17
GO:0006953	acute-phase response	3089	9	1	2923	3102	0.00973	9.55
GO:0008535	respiratory chain complex IV assembly	3087	11	2	2914	3101	0.0108	5.85
GO:0010575	positive regulation of vascular endothelial growth factor production	3080	6	0	2903	3099	0.0129	inf
GO:0003012	muscle system process	3064	13	32	2897	3099	0.0104	0.435
GO:0035094	response to nicotine	2995	6	0	2884	3067	0.013	inf
GO:0071774	response to fibroblast growth factor	2988	6	0	2878	3067	0.0129	inf

GO:0007212	dopamine receptor signaling pathway	2981	0	7	2872	3067	0.0159	0
GO:0018198	peptidyl-cysteine modification	2976	0	7	2872	3060	0.0159	0
GO:0043457	regulation of cellular respiration	2972	8	1	2872	3053	0.0182	8.5
GO:0046822	regulation of nucleocytoplasmic transport	2959	4	17	2864	3052	0.00777	0.251
GO:0007268	chemical synaptic transmission	2916	9	23	2860	3035	0.0217	0.415
GO:0022406	membrane docking	2891	19	6	2851	3012	0.00801	3.35
GO:0050796	regulation of insulin secretion	2861	6	21	2832	3006	0.00649	0.303
GO:0098656	anion transmembrane transport	2831	28	12	2826	2985	0.0102	2.46
GO:0070873	regulation of glycogen metabolic process	2819	6	0	2798	2973	0.013	inf
GO:0042391	regulation of membrane potential	2816	10	26	2792	2973	0.018	0.41
GO:0036293	response to decreased oxygen levels	2771	29	12	2782	2947	0.00468	2.56
GO:0001909	leukocyte mediated cytotoxicity	2713	6	0	2753	2935	0.0129	inf
GO:0071478	cellular response to radiation	2705	14	4	2747	2935	0.0163	3.74
GO:0045834	positive regulation of lipid metabolic process	2685	8	1	2733	2931	0.0178	8.58
GO:0030168	platelet activation	2674	8	1	2725	2930	0.0177	8.6
GO:0030101	natural killer cell activation	2667	1	9	2717	2929	0.0223	0.12
GO:0048513	animal organ development	2649	85	128	2716	2920	0.0176	0.714
GO:0009887	animal organ morphogenesis	2406	9	27	2631	2792	0.00659	0.354
GO:0031214	biomineral tissue development	2374	9	1	2622	2765	0.00992	9.49
GO:0016338	calcium-independent cell-cell adhesion via plasma membrane cell-adhesion molecules	2364	1	10	2613	2764	0.012	0.106
GO:0050921	positive regulation of chemotaxis	2359	2	12	2612	2754	0.0133	0.176
GO:0071219	cellular response to molecule of bacterial origin	2331	19	7	2610	2742	0.017	2.85
GO:0051653	spindle localization	2302	8	1	2591	2735	0.0186	8.44
GO:2001243	negative regulation of intrinsic apoptotic signaling pathway	2286	1	9	2583	2734	0.0219	0.118
GO:0097191	extrinsic apoptotic signaling pathway	2279	12	3	2582	2725	0.0185	4.22
GO:0051704	multi-organism process	2267	60	93	2570	2722	0.0264	0.683
GO:0045444	fat cell differentiation	2123	5	17	2510	2629	0.0174	0.308
GO:0022409	positive regulation of cell-cell adhesion	2111	10	25	2505	2612	0.0174	0.417
GO:0120031	plasma membrane bounded cell projection assembly	2055	25	11	2495	2587	0.0183	2.36
GO:0006997	nucleus organization	2012	10	2	2470	2576	0.0201	5.21
GO:0072376	protein activation cascade	2003	13	4	2460	2574	0.0278	3.4

GO:0032869	cellular response to insulin stimulus	1994	5	0	2447	2570	0.0277	inf
GO:0002824	positive regulation of adaptive immune response based on somatic recombination of immune receptors built from immunoglobulin superfamily domains	1989	5	0	2442	2570	0.0275	inf
GO:0001942	hair follicle development	1970	5	0	2437	2570	0.0274	inf
GO:0014910	regulation of smooth muscle cell migration	1964	5	0	2432	2570	0.0273	inf
GO:0003279	cardiac septum development	1957	5	0	2427	2570	0.0271	inf
GO:0007006	mitochondrial membrane organization	1954	16	6	2422	2570	0.0308	2.83
GO:0043086	negative regulation of catalytic activity	1945	19	37	2406	2564	0.0319	0.547
GO:0043279	response to alkaloid	1914	7	1	2387	2527	0.0343	7.41
GO:0002790	peptide secretion	1898	10	2	2380	2526	0.0193	5.31
GO:1901701	cellular response to oxygen-containing compound	1887	11	25	2370	2524	0.0432	0.469
GO:1901700	response to oxygen-containing compound	1844	29	16	2359	2499	0.0363	1.92
GO:0009607	response to biotic stimulus	1785	0	7	2330	2483	0.0159	0
GO:0035036	sperm-egg recognition	1780	0	6	2330	2476	0.0316	0
GO:0038127	ERBB signaling pathway	1777	9	2	2330	2470	0.0339	4.77
GO:0007186	G-protein coupled receptor signaling pathway	1766	43	68	2321	2468	0.0439	0.672
GO:0070085	glycosylation	1716	33	55	2278	2400	0.0407	0.632
GO:1901135	carbohydrate derivative metabolic process	1687	41	21	2245	2345	0.0072	2.04
GO:0031348	negative regulation of defense response	1649	9	2	2204	2324	0.0343	4.75
GO:0030030	cell projection organization	1640	30	51	2195	2322	0.0433	0.622
GO:0045773	positive regulation of axon extension	1570	5	0	2165	2271	0.0278	inf
GO:0051094	positive regulation of developmental process	1562	32	56	2160	2271	0.0235	0.601
GO:0060395	SMAD protein signal transduction	1469	6	0	2128	2215	0.0139	inf
GO:0043666	regulation of phosphoprotein phosphatase activity	1466	5	0	2122	2215	0.0281	inf
GO:0040017	positive regulation of locomotion	1456	16	6	2117	2215	0.0314	2.79
GO:0031032	actomyosin structure organization	1427	2	11	2101	2209	0.0229	0.191
GO:0006885	regulation of pH	1410	7	1	2099	2198	0.0352	7.33
GO:0006897	endocytosis	1402	51	33	2092	2197	0.0358	1.62
GO:0016050	vesicle organization	1374	10	25	2041	2164	0.0259	0.424
GO:0032434	regulation of proteasomal ubiquitin-dependent protein catabolic process	1353	14	5	2031	2139	0.0372	2.95
GO:0006913	nucleocytoplasmic transport	1344	32	18	2017	2134	0.0329	1.88

GO:0051668	localization within membrane	1325	2	10	1985	2116	0.0396	0.213
GO:1904062	regulation of cation transmembrane transport	1310	1	8	1983	2106	0.0397	0.133
GO:0048468	cell development	1298	27	15	1982	2098	0.0447	1.91
GO:0045665	negative regulation of neuron differentiation	1277	5	0	1955	2083	0.0267	inf
GO:0006575	cellular modified amino acid metabolic process	1267	7	18	1950	2083	0.0459	0.415
GO:0070527	platelet aggregation	1254	6	1	1943	2065	0.0628	6.38
GO:0006413	translational initiation	1250	5	14	1937	2064	0.0658	0.381
GO:0010628	positive regulation of gene expression	1249	16	7	1932	2050	0.0582	2.43

Top gene ontology categories (FDR <= 5%) for significantly differentially expressed genes in [rAAV:HDR:cleaved vs rAAV:FahDonor:ncSpacer]. All tests were done using an iterative gene ontology analysis (Methods). GO.NAMESPACE biological_process

GO.ID	GO.NAME	N.TEST	CAT.N.FG.GENE	CAT.N.BG.GENE	N.FG.GENE	N.BG.GENE	P	ODDS.RATIO
GO:0044281	small molecule metabolic process	6365	538	438	4594	5473	1.54E-08	1.46
GO:0010951	negative regulation of endopeptidase activity	5556	70	39	4056	5035	5.88E-05	2.23
GO:0070527	platelet aggregation	5495	24	7	3986	4996	0.000223	4.3
GO:0045475	locomotor rhythm	5477	10	0	3962	4989	0.000291	inf
GO:0006412	translation	5470	98	70	3952	4989	0.000295	1.77
GO:0007600	sensory perception	5436	45	107	3854	4919	0.000369	0.537
GO:0060487	lung epithelial cell differentiation	5310	0	13	3809	4812	0.00093	0
GO:1903035	negative regulation of response to wounding	5283	22	7	3809	4799	0.00104	3.96
GO:0044380	protein localization to cytoskeleton	5237	17	5	3787	4792	0.00211	4.3
GO:0006629	lipid metabolic process	5217	140	120	3770	4787	0.00188	1.48
GO:0040029	regulation of gene expression, epigenetic	5040	20	57	3630	4667	0.00169	0.451
GO:0007272	ensheathment of neurons	4983	4	23	3610	4610	0.00278	0.222
GO:0006623	protein targeting to vacuole	4966	0	10	3606	4587	0.00331	0
GO:0031290	retinal ganglion cell axon guidance	4958	0	10	3606	4577	0.00328	0
GO:0008535	respiratory chain complex IV assembly	4937	11	2	3606	4567	0.00396	6.97
GO:0033555	multicellular organismal response to stress	4930	4	21	3595	4565	0.00425	0.242
GO:0035136	forelimb morphogenesis	4899	0	9	3591	4544	0.00594	0
GO:0032963	collagen metabolic process	4879	2	15	3591	4535	0.00643	0.168
GO:0006120	mitochondrial electron transport, NADH to ubiquinone	4863	6	0	3589	4520	0.00754	inf
GO:1900040	regulation of interleukin-2 secretion	4862	6	0	3583	4520	0.0075	inf
GO:0030514	negative regulation of BMP signaling pathway	4851	3	19	3577	4520	0.00413	0.2
GO:0090092	regulation of transmembrane receptor protein serine/threonine kinase signaling pathway	4802	52	33	3574	4501	0.00202	1.98
GO:1903169	regulation of calcium ion transmembrane transport	4669	10	37	3522	4468	0.00169	0.343
GO:0007140	male meiotic nuclear division	4593	2	16	3512	4431	0.00385	0.158
GO:0034587	piRNA metabolic process	4581	11	1	3510	4415	0.000925	13.8
GO:0050873	brown fat cell differentiation	4575	13	3	3499	4414	0.00405	5.47
GO:0009887	animal organ morphogenesis	4561	61	123	3486	4411	0.00328	0.628
GO:0038127	ERBB signaling pathway	4261	16	5	3425	4288	0.00385	4.01
GO:1901216	positive regulation of neuron death	4251	3	18	3409	4283	0.00678	0.209
GO:0006812	cation transport	4210	147	132	3406	4265	0.00693	1.39

GO:0051260	protein homooligomerization	4040	51	35	3259	4133	0.00604	1.85
GO:0035924	cellular response to vascular endothelial growth factor stimulus	3969	6	0	3208	4098	0.00719	inf
GO:0006370	7-methylguanosine mRNA capping	3954	6	0	3202	4098	0.00715	inf
GO:0007219	Notch signaling pathway	3946	9	30	3196	4098	0.00923	0.385
GO:0035282	segmentation	3912	10	2	3187	4068	0.00739	6.38
GO:0048534	hematopoietic or lymphoid organ development	3894	14	43	3177	4066	0.00307	0.417
GO:0007030	Golgi organization	3807	10	33	3163	4023	0.00801	0.385
GO:0071407	cellular response to organic cyclic compound	3786	45	31	3153	3990	0.0103	1.84
GO:0002790	peptide secretion	3642	25	13	3108	3959	0.00828	2.45
GO:0045744	negative regulation of G-protein coupled receptor protein signaling pathway	3587	0	8	3083	3946	0.0113	0
GO:0006312	mitotic recombination	3582	0	8	3083	3938	0.0112	0
GO:0001894	tissue homeostasis	3575	24	13	3083	3930	0.0124	2.35
GO:0046677	response to antibiotic	3539	8	1	3059	3917	0.013	10.2
GO:0010574	regulation of vascular endothelial growth factor production	3531	8	1	3051	3916	0.013	10.3
GO:0045664	regulation of neuron differentiation	3505	90	79	3043	3915	0.015	1.47
GO:2000648	positive regulation of stem cell proliferation	3338	11	2	2953	3836	0.00359	7.14
GO:0045324	late endosome to vacuole transport	3313	5	0	2942	3834	0.0155	inf
GO:0051310	metaphase plate congression	3306	3	17	2937	3834	0.0115	0.23
GO:0002888	positive regulation of myeloid leukocyte mediated immunity	3292	5	0	2934	3817	0.0155	inf
GO:2001242	regulation of intrinsic apoptotic signaling pathway	3275	10	31	2929	3817	0.0168	0.42
GO:0043618	regulation of transcription from RNA polymerase II promoter in response to stress	3223	5	0	2919	3786	0.0157	inf
GO:0009060	aerobic respiration	3219	0	7	2914	3786	0.0214	0
GO:0071560	cellular response to transforming growth factor beta stimulus	3215	0	7	2914	3779	0.0213	0
GO:0018108	peptidyl-tyrosine phosphorylation	3205	25	15	2914	3772	0.024	2.16
GO:0043567	regulation of insulin-like growth factor receptor signaling pathway	3181	7	1	2889	3757	0.0249	9.1
GO:0043279	response to alkaloid	3179	8	2	2882	3756	0.025	5.21
GO:0002755	MyD88-dependent toll-like receptor signaling pathway	3163	5	0	2874	3754	0.0154	inf
GO:0001885	endothelial cell development	3154	0	7	2869	3754	0.0217	0
GO:0003279	cardiac septum development	3148	8	2	2869	3747	0.0248	5.22
GO:0003341	cilium movement	3135	4	17	2861	3745	0.0271	0.308

GO:0032147	activation of protein kinase activity	3129	12	33	2857	3728	0.0239	0.474
GO:0030032	lamellipodium assembly	3094	10	3	2845	3695	0.0221	4.33
GO:0048608	reproductive structure development	3081	9	27	2835	3692	0.0281	0.434
GO:0014068	positive regulation of phosphatidylinositol 3-kinase signaling	3043	8	2	2826	3665	0.0253	5.19
GO:0007178	transmembrane receptor protein serine/threonine kinase signaling pathway	3037	4	17	2818	3663	0.0268	0.306
GO:0045599	negative regulation of fat cell differentiation	3020	1	10	2814	3646	0.029	0.13
GO:0080135	regulation of cellular response to stress	3006	59	50	2813	3636	0.032	1.53
GO:0009100	glycoprotein metabolic process	2884	16	7	2754	3586	0.0184	2.98
GO:1903320	regulation of protein modification by small protein conjugation or removal	2872	19	46	2738	3579	0.0234	0.54
GO:0031647	regulation of protein stability	2820	40	29	2719	3533	0.0198	1.79
GO:0007212	dopamine receptor signaling pathway	2759	0	8	2679	3504	0.0119	0
GO:0045598	regulation of fat cell differentiation	2756	13	6	2679	3496	0.0358	2.83
GO:0070661	leukocyte proliferation	2741	13	6	2666	3490	0.0356	2.84
GO:1904950	negative regulation of establishment of protein localization	2717	8	26	2653	3484	0.0232	0.404
GO:1903828	negative regulation of cellular protein localization	2690	9	2	2645	3458	0.0131	5.88
GO:0034104	negative regulation of tissue remodeling	2681	5	0	2636	3456	0.0152	inf
GO:0043248	proteasome assembly	2673	8	2	2631	3456	0.0244	5.25
GO:0048863	stem cell differentiation	2669	1	10	2623	3454	0.0294	0.132
GO:0009653	anatomical structure morphogenesis	2660	62	115	2622	3444	0.031	0.708
GO:0071695	anatomical structure maturation	2504	9	3	2560	3329	0.0387	3.9
GO:0007292	female gamete generation	2495	9	3	2551	3326	0.0386	3.91
GO:0002825	regulation of T-helper 1 type immune response	2488	0	6	2542	3323	0.0397	0
GO:0018198	peptidyl-cysteine modification	2477	0	6	2542	3317	0.0396	0
GO:0072376	protein activation cascade	2475	14	7	2542	3311	0.045	2.61
GO:0009581	detection of external stimulus	2461	7	2	2528	3304	0.0462	4.57
GO:0031032	actomyosin structure organization	2457	3	15	2521	3302	0.0296	0.262
GO:0072507	divalent inorganic cation homeostasis	2445	8	25	2518	3287	0.0332	0.418
GO:0007186	G-protein coupled receptor signaling pathway	2428	44	86	2510	3262	0.0314	0.665
GO:0002673	regulation of acute inflammatory response	2361	5	0	2466	3176	0.016	inf
GO:0002250	adaptive immune response	2357	10	28	2461	3176	0.0331	0.461

GO:0019932	second-messenger-mediated signaling	2317	7	1	2451	3148	0.0251	8.99
GO:1900542	regulation of purine nucleotide metabolic process	2308	6	0	2444	3147	0.00701	inf
GO:0009628	response to abiotic stimulus	2306	67	59	2438	3147	0.0367	1.47
GO:0019827	stem cell population maintenance	2151	3	16	2371	3088	0.0184	0.244
GO:0001822	kidney development	2137	1	10	2368	3072	0.029	0.13
GO:0051100	negative regulation of binding	2123	10	3	2367	3062	0.0222	4.31
GO:0071900	regulation of protein serine/threonine kinase activity	2112	20	11	2357	3059	0.0277	2.36
GO:0097028	dendritic cell differentiation	2089	0	6	2337	3048	0.0396	0
GO:0006953	acute-phase response	2078	7	2	2337	3042	0.0467	4.56
GO:0006607	NLS-bearing protein import into nucleus	2074	7	2	2330	3040	0.0464	4.57
