# Supplementary material for: Self-inactivating, all-in-one AAV vectors for precision Cas9 genome editing via homology-directed repair in vivo
Source: Nat Commun. 2021 Nov 1;12:6267. doi: 10.1038/s41467-021-26518-y (PMC8560862; doi:10.1038/s41467-021-26518-y)
Supplement: Supplementary file 3 — Reporting Summary [file 41467_2021_26518_MOESM3_ESM.pdf]

nature research

Corresponding author(s): Erik J. Sontheimer

Last updated by author(s): Sep 27, 2021

Reporting Summary

Nature Research wishes to improve the reproducibility of the work that we publish. This form provides structure for consistency and transparency in reporting. For further information on Nature Research policies, see our Editorial Policies and the Editorial Policy Checklist.

Statistics

For all statistical analyses, confirm that the following items are present in the figure legend, table legend, main text, or Methods section.

n/a	Confirmed	
☐	☒	The exact sample size (n) for each experimental group/condition, given as a discrete number and unit of measurement
☐	☒	A statement on whether measurements were taken from distinct samples or whether the same sample was measured repeatedly
☐	☒	The statistical test(s) used AND whether they are one- or two-sided<br>*Only common tests should be described solely by name; describe more complex techniques in the Methods section.*
☒	☐	A description of all covariates tested
☐	☒	A description of any assumptions or corrections, such as tests of normality and adjustment for multiple comparisons
☐	☒	A full description of the statistical parameters including central tendency (e.g. means) or other basic estimates (e.g. regression coefficient) AND variation (e.g. standard deviation) or associated estimates of uncertainty (e.g. confidence intervals)
☐	☒	For null hypothesis testing, the test statistic (e.g. F, t, r) with confidence intervals, effect sizes, degrees of freedom and P value noted<br>*Give P values as exact values whenever suitable.*
☒	☐	For Bayesian analysis, information on the choice of priors and Markov chain Monte Carlo settings
☒	☐	For hierarchical and complex designs, identification of the appropriate level for tests and full reporting of outcomes
☒	☐	Estimates of effect sizes (e.g. Cohen's d, Pearson's r), indicating how they were calculated

Our web collection on statistics for biologists contains articles on many of the points above.

Software and code

Policy information about availability of computer code

Data collection	FACS data was collected using a MACSQuant® VYB from Miltenyi Biotec. Long-read SMRT sequencing data were collected using a Pacific Biosciences Sequel II. Next-generation targeted amplicon sequencing data were generated using an Illumina MiSeq (Fah locus) or MiniSeq (Idua locus), while RNA-Seq was collected using an Illumina NextSeq 500. Quantitative PCR and qRT-PCR data were collected using QuantStudio Design and Analysis Desktop Software V1.5.1 on a QuantStudio 3 Real-Time PCR System (Applied biosystems). The absorbance at 450 nm was measured using a BioTek Synergy HT microplate reader (BioTek) and the Gen5 software. AST and ALT levels were determined using Cobas Clinical Chemistry Analyzer (RRID:SCR_015365). LAMP-1 western blot was scanned using LI-COR scanner (Odyssey).
Data analysis	TIDE web tool (https://tide-calculator.nki.nl/); Microsoft Excel Version 1902 (Build 11328.20644); GraphPad Prism 8.4.3; CRISPResso 2.0.40; BEDTools (version 2.27.0); Kallisto v0.45.0; STAR v2.7.5a; HTSeq v.0.10.0; DESeq2 release 3.11; SMRT Link v8; BWA-MEM (Galaxy Version 0.7.17.1); fastp (Galaxy Version 0.20.1+galaxy0); Cutadapt (Galaxy Version 1.16.6); ccs (smrtlink/8.0.0.79519); Galaxy web platform at usegalaxy.org (20.09.rc1); FASTQ/A Barcode Splitter (Galaxy Version 1.0.1); UCLUST (usearch v7.0.1090); CRISPRseek (Version 1.32.0); UDiTaS v1.0; DolphinNext RNA-seq pipeline (revision 4); RSEM (v1.3.1); DESeq2 software (v 1.28.1); Image Studio Lite (LI-COR); MinKNOW software (v19.10.1); Biorender.com.

For manuscripts utilizing custom algorithms or software that are central to the research but not yet described in published literature, software must be made available to editors and reviewers. We strongly encourage code deposition in a community repository (e.g. GitHub). See the Nature Research guidelines for submitting code & software for further information.

Data

Policy information about availability of data

All manuscripts must include a data availability statement. This statement should provide the following information, where applicable:

- Accession codes, unique identifiers, or web links for publicly available datasets
- A list of figures that have associated raw data
- A description of any restrictions on data availability

All sequencing data that support the findings of this study have been deposited in the NIH Sequence Read Archive via BioProject PRJNA667456. Source data are provided with this paper.

Field-specific reporting

Please select the one below that is the best fit for your research. If you are not sure, read the appropriate sections before making your selection.

☒ Life sciences ☐ Behavioural & social sciences ☐ Ecological, evolutionary & environmental sciences

For a reference copy of the document with all sections, see nature.com/documents/nr-reporting-summary-flat.pdf

Life sciences study design

All studies must disclose on these points even when the disclosure is negative.

Sample size	Samples sizes were determined based on our and others' previous work that generated reproducible results (Pankowicz, F. et al, Nat Comm, 2016; Wang, D. et al, Nat Biotechnol, 2018; Song, Q., Nat Biomed Eng, 2020)
Data exclusions	Two negative-control hereditary tyrosinemia Fah (PM/PM) mice injected with PBS and rAAV:ncDonor:FahSpacer were excluded from (Supplementary Fig. 3d and e) as the sera from these mice were hemolyzed and readouts of AST and ALT were not possible. One biological replicate of plasmid transfection of Dual-sgRNA:Design 1 and 4 failed SMRT sequencing and was excluded from analysis.
Replication	All of the in vitro cell culture CRISPR editing experiments were performed in replicates independently and in separate days as indicated in the samples size (n) in figure legends. The successful creation of the molecular and/or physiological phenotypes in the HT-I and MPS-I mice before treatment, and the amelioration of those phenotypes after successful gene editing confirm that the findings of our study are reproducible following the standards of the field. All standard deviations were in the expected ranges.
Randomization	Samples were randomly assigned between control and treatment groups.
Blinding	Samples were prepared unblinded, and under same conditions between different treatment groups. Blinding was not relevant in this study as Cas9-based in vivo genome editing has been previously established by independent studies in the HT-I and MPS-I mouse models (Yin, H., Nat Biotechnol. 2014; Schuh, R., Gene Ther., 2020; Wang, D. et al, Nat Biotechnol, 2018). Quantification of editing efficiency and other parameters for physiological improvements of phenotypes were applied similarly across all treatment groups and replicates.

Reporting for specific materials, systems and methods

We require information from authors about some types of materials, experimental systems and methods used in many studies. Here, indicate whether each material, system or method listed is relevant to your study. If you are not sure if a list item applies to your research, read the appropriate section before selecting a response.

Materials & experimental systems

n/a	Involved in the study
☐	☒ Antibodies
☐	☒ Eukaryotic cell lines
☒	☐ Palaeontology and archaeology
☐	☒ Animals and other organisms
☒	☐ Human research participants
☒	☐ Clinical data
☒	☐ Dual use research of concern

Methods

n/a	Involved in the study
☒	☐ ChIP-seq
☐	☒ Flow cytometry
☒	☐ MRI-based neuroimaging

Antibodies

Antibodies used	Anti-HPD (Sigma HPA038322, dilution: 1:600 for Western blot & 1:40 for IHC). Horseradish peroxidase (HRP)-conjugated goat anti-rabbit (Bio-Rad 1706515, 1:10,000 dilution). Rabbit anti-GAPDH (Abcam ab9485, dilution 1:2,000). Anti-FAH antibody (Abcam, Cat. No. ab81087; dilution 1:400). Rat anti-LAMP1 (BD Pharmingen, RUO - 553792; dilution 1:2000). IRDye® 680RD Goat anti-Rat IgG secondary antibody (LI-COR Biosciences, 926-68076; dilution 1:5000)

Validation	- Anti-HPD (Sigma HPA038322) was not independently validated, instead; we followed the manufacturer's recommendation (https://www.sigmaaldrich.com/catalog/product/sigma/hpa038322?lang=en&region=US)<br>- Horseradish peroxidase (HRP)-conjugated goat anti-rabbit (Bio-Rad 1706515): we validated and used this antibody in a previous publication (https://rdcu.be/b72Nz)<br>- Rabbit anti-GAPDH (Abcam ab9485): we validated and used this antibody in a previous publication (https://rdcu.be/b72Nz)<br>- Anti-FAH antibody (Abcam, Cat. No. ab81087): We validated it in a previous publication (https://www.ncbi.nlm.nih.gov/pmc/articles/PMC6126964/)<br>- Rat anti-LAMP1 (BD Pharmingen, RUO - 553792) and IRDye® 680RD Goat anti-Rat IgG secondary antibody (LI-COR Biosciences, 926-68076) were validated by the manufacturer. Appropriate controls of wild-type and MPS-I untreated (non-cognate spacer injected) mice were used was positive and negative controls.

Eukaryotic cell lines

Policy information about cell lines

Cell line source(s)	Cell line: HEK293T; Source: ATCC (ATCC® CRL-3216™)<br>Cell line: Neuro2a; Source: ATCC (ATCC® CCL-131™)<br>Cell line: MOLT-3; Source: ATCC (ATCC® CRL-1552™)<br>Cell line: TLR-MCV1 HEK293T; Source (Dr. Erik Sontheimer - Univ. Massachusetts Medical School; as described in a previous preprint in revision at a different journal (https://www.biorxiv.org/content/10.1101/864199v1.full)
Authentication	Not authenticated.
Mycoplasma contamination	Not tested.
Commonly misidentified lines (See ICLAC register)	No commonly misidentified cell line was used.

Animals and other organisms

Policy information about studies involving animals; ARRIVE guidelines recommended for reporting animal research

Laboratory animals	All mice were housed in the animal facility at the University of Massachusetts Medical School with ambient temperature of 65-75°F (~18-23°C), 40-60% humidity and on a 14-hour light/10-hour dark cycle.<br>- HT-I mice were kindly provided by Dr. M. Grompe and maintained on C57 background for the Fah(PM/PM) strain, and on a 129 background for Fah(neo/neo) strain. Both male and female mice (ages: 8 to 15 weeks old) were used.<br>- Homozygous MPS-I Idua(W392X) mice were purchased from the Jackson Laboratory (Stock No. 017681) and used to breed neonatal Idua(W392X) pups for rAAV injections. Both male and female neonate mice (ages: 1 day old) were used.<br>- C57Bl/6 mice were purchased from the Jackson Laboratory (Stock No. 000664). Both male and female mice (ages: 8 to 15 weeks old) were used.
Wild animals	The study did not involve wild animals.
Field-collected samples	The study did not involve samples collected in the field.
Ethics oversight	All animal study procedures were approved by the Institutional Animal Care and Use Committee (IACUC) at the University of Massachusetts Medical School.

Note that full information on the approval of the study protocol must also be provided in the manuscript.

Flow Cytometry

Plots

Confirm that:

- [x] The axis labels state the marker and fluorochrome used (e.g. CD4-FITC).
- [x] The axis scales are clearly visible. Include numbers along axes only for bottom left plot of group (a 'group' is an analysis of identical markers).
- [x] All plots are contour plots with outliers or pseudocolor plots.
- [x] A numerical value for number of cells or percentage (with statistics) is provided.

Methodology

Sample preparation	Cells were transfected as described in Methods. After 72 hours, the cells were trypsinized and washed with PBS twice, before analyzing on a MACSQuant VYB.
Instrument	MACSQuant® VYB - Miltenyi Biotec
Software	Flowjo V.9.0

Cell population abundance	The abundance of cells after gating was dependent on the design of the AAV construct being tested. >70% of the cells passed the "singlets" gate. The mCherry+ efficiencies ranged from 0-30% and GFP+ efficiencies ranged from 0-5%.
Gating strategy	Cells were first gated based on forward and side scatter to select "live" cells. Live cells were further selected for singlets based on FSC-H and FSC-A. This population of cells was then gated for mCherry and GFP to measure indels and HDR rates respectively. The boundaries of mCherry and GFP gates were selected based on the negative (unedited) controls.

☒ Tick this box to confirm that a figure exemplifying the gating strategy is provided in the Supplementary Information.
